# Supplementary material for: SNHG10 promotes tumorigenesis through the EGFR/AKT/ERK/mTOR and miR-150-5p/VEGF-A axis, along with gemcitabine resistance in pancreatic ductal adenocarcinoma
Source: Cell Death Discov. 2026 Mar 31;12:210. doi: 10.1038/s41420-026-03040-y (PMC13161215; doi:10.1038/s41420-026-03040-y)
Supplement: Supplementary file 1 — Supplementary Material [file 41420_2026_3040_MOESM1_ESM.docx]

**SNHG10 promotes tumorigenesis through the EGFR/AKT/ERK/mTOR and miR-150-5p/VEGF-A axis, along with gemcitabine resistance in pancreatic ductal adenocarcinoma**

**Gouri Pandya^1^, Aishwarya Singh^1^, Suman Saurav^2^, Sharon Raju^2^, Rachana Kumari^1^, Rashi Sharma^3^, Shinjinee Sengupta^1^, Vidhi Goyal^4^, Bhudev C Das^1^, Gautam Sethi^5^, Amit Kumar Pandey^6^, Deepti Pandita^7^, Rajender K Motiani^2^, Manoj Garg^1,^***

^1^Amity Institute of Molecular Medicine and Stem Cell Research (AIMMSCR), Amity University, Uttar Pradesh, Sector-125, Noida-201313, India

^2^Laboratory of Calciomics and Systemic Pathophysiology (LCSP), Regional Centre for Biotechnology (RCB), Faridabad-121001, India

^3^Department of Histopathology, Pathology and Laboratory Medicine, Medanta Hospital, Gurgaon, India

^4^Amity Foundation for Science, Technology and Innovation Alliances, Amity University, Uttar Pradesh, Sector-125, Noida-201313, India

^5^Department of Pharmacology, Yong Loo Lin School of Medicine, National University of Singapore, Singapore, 117600, Singapore.

^6^Department of Biotechnology, National Institute of Pharmaceutical Education & Research (NIPER) Ahmedabad, Gandhinagar-382355, India

^7^Delhi Institute of Pharmaceutical Sciences & Research (DIPSAR), Delhi Pharmaceutical Sciences & Research University (DPSRU), New Delhi-110017, India

***Corresponding author:** Dr. Manoj Garg, Amity Institute of Molecular Medicine and Stem Cell Research (AIMMSCR), Amity University Uttar Pradesh, Sector-125, Noida-201313, India.

**E-mail address:** mgarg@amity.edu; nuscsimg@gmail.com

**SUPPLEMENTARY FIGURES**

**
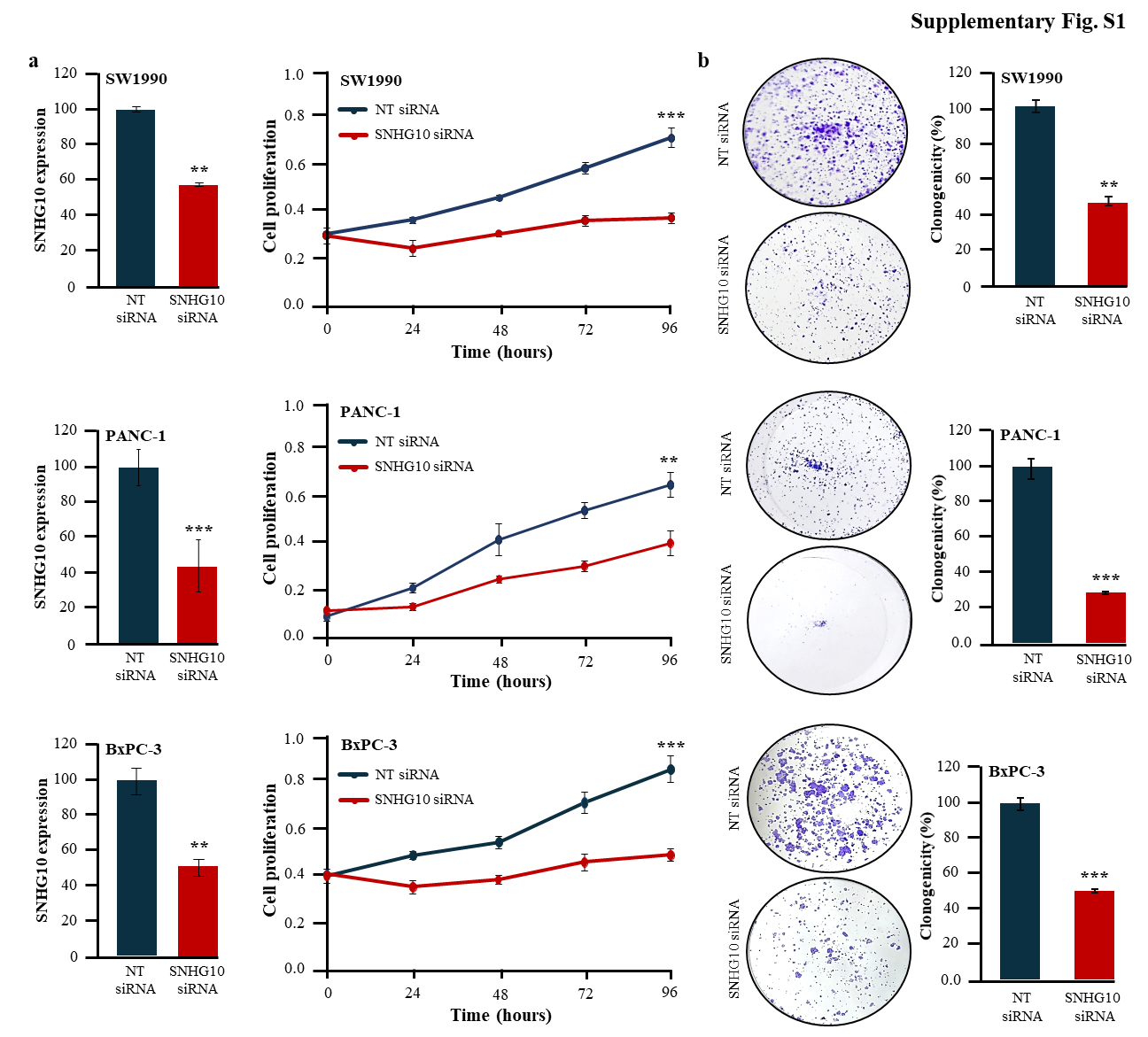
Supplementary Fig. S1. Depletion of SNHG10 significantly suppresses the viability and clonogenicity of PDAC cells.** (a) The qRT-PCR displayed the depletion of SNHG10 in siRNA-transfected SW1990, PANC-1, and BxPC-3 cell lines. MTT assay measures the proliferation ability of SW1990, PANC-1, and BxPC-3 cells transfected with SNHG10 siRNA. (b) Colony formation assay confirmed clonogenicity of SNHG10 siRNA-transfected PDAC cells. All the experiments were performed three times in biological triplicate. The results are presented as the means ± SDs; n=3. *p < 0.05, **p < 0.01, ***p < 0.001; two-tailed Student t test.


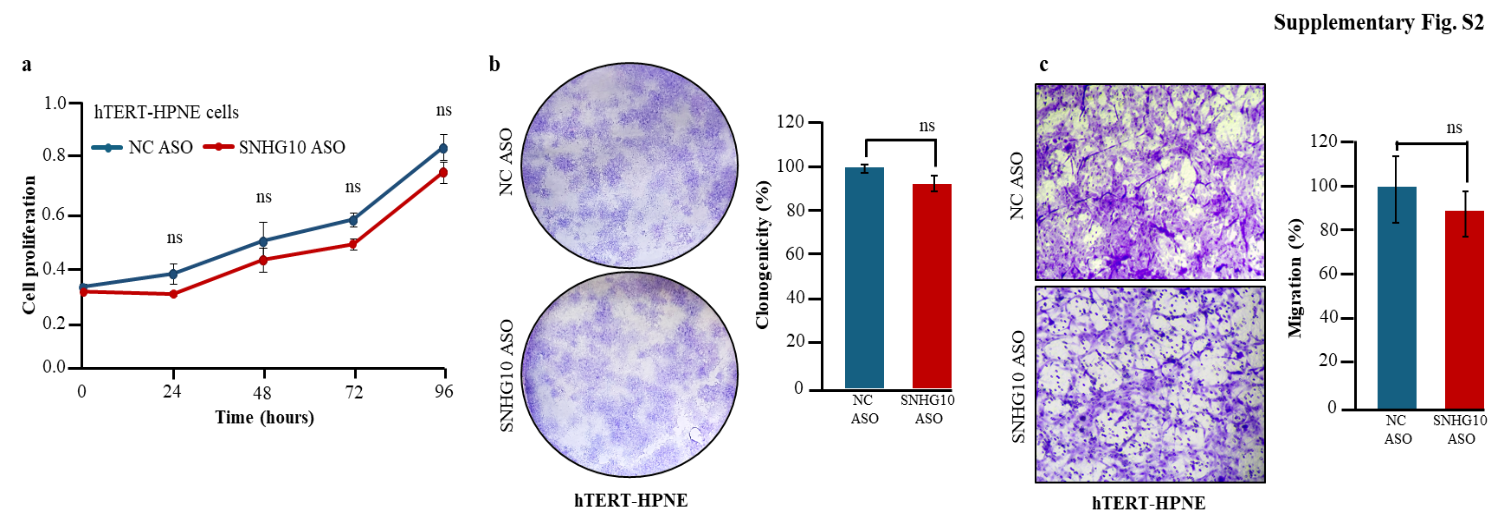


**Supplementary Fig. S2 Effect of SNHG10 depletion on cell viability, clonogenicity, and migration of human normal pancreatic epithelial cells.** (a) The cell viability was confirmed by MTT assay. (b) Clonogenicity measured the colony-forming capacity of hTERT-HPNE. (c) Boyden chamber assay determined the migration ability of hTERT-HPNE cells. Student t-test was performed for statistical significance. ns; not significant.


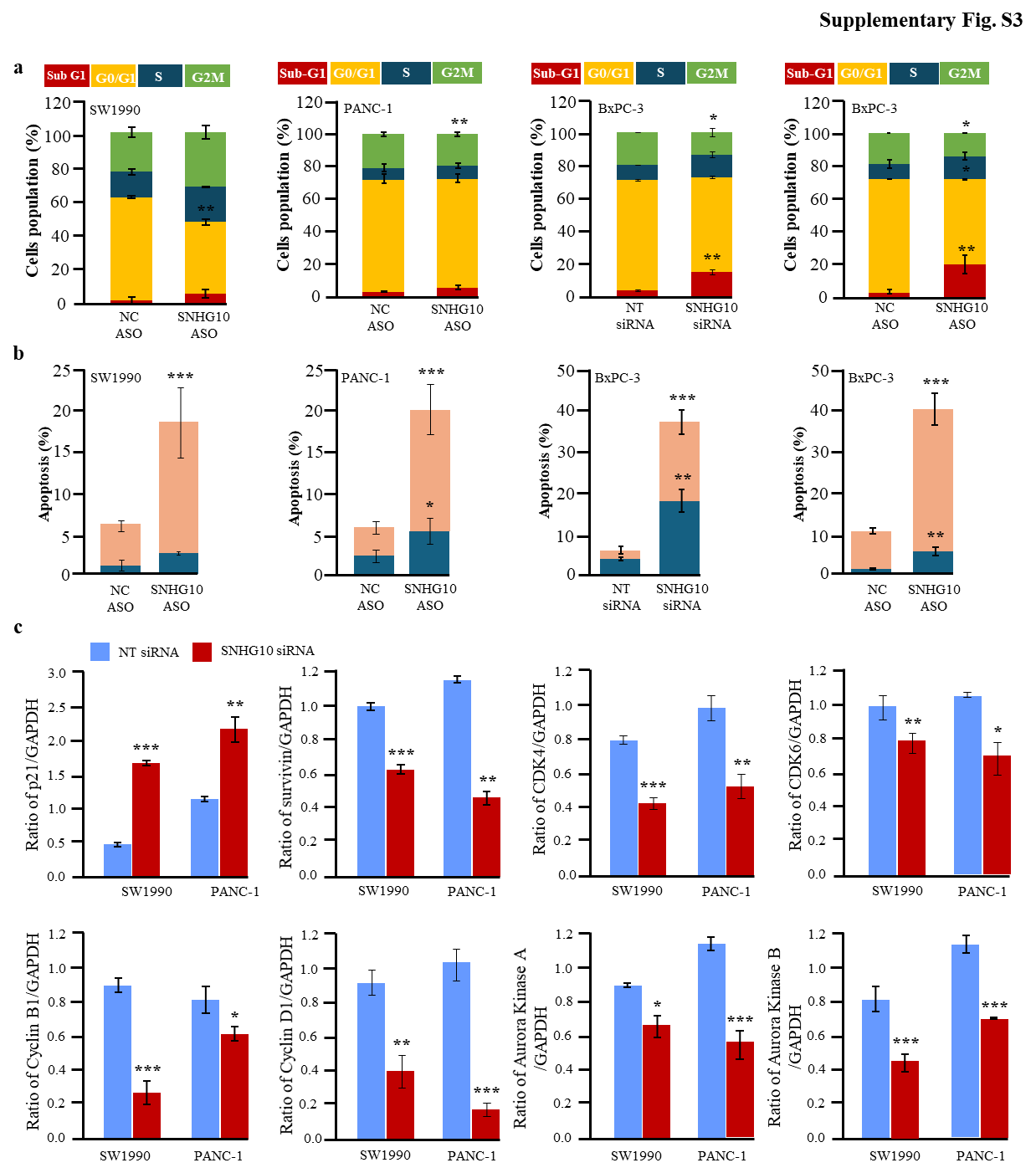


**Supplementary Fig. S3. SNHG10 depletion induced cell cycle arrest and apoptosis**. (a) Cell cycle distribution measured by propidium iodide staining in SNHG10-depleted SW1990, PANC-1, and BxPC-3 cells. Quantification and histogram represented the cell population in different phases of the cell cycle. (b) Cell apoptosis was determined using Annexin V and propidium iodide staining in SNHG10-depleted SW1990, PANC-1, and BxPC-3 cells. (c) Densitometry of Western blots of SNHG10-silenced PDAC cell lines displayed a significant difference in the expression of p21, survivin, CDK4, CDK6, cyclin B1, cyclin D1, arora kinase A, and arora kinase B proteins. The results are presented as the means ± SDs; n=3. *p < 0.05, **p < 0.01, ***p < 0.001; two-tailed Student t test.

**
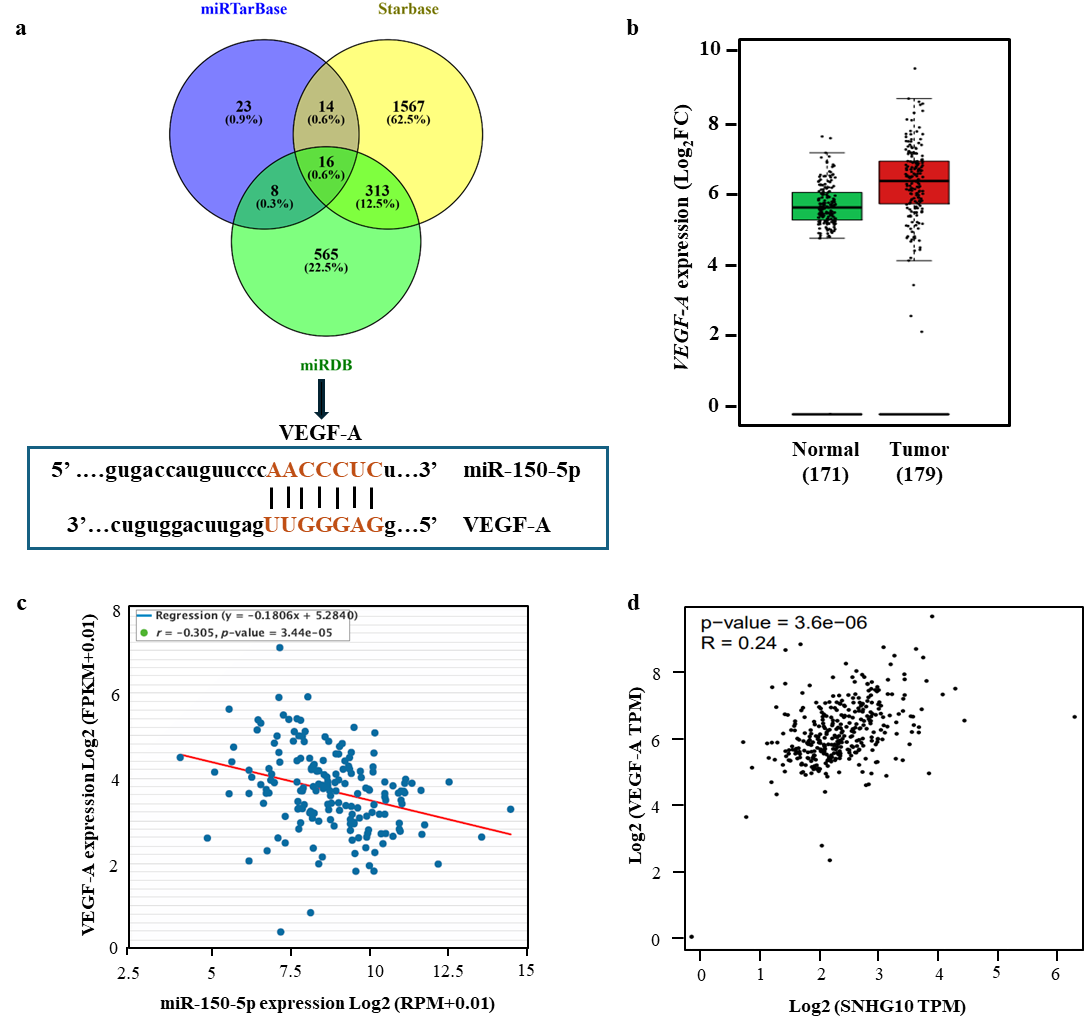
**

**Supplementary Fig. S4. SNHG10 interacted with miR-150-5p and modulated the expression of VEGF-A in PDAC.** (a) The miRTarbase, starbase, and miRDB databases predicted VEGF-A as the target gene of miR‐50‐5p, as shown by the Venn diagram, and conservation in binding among miR‐150‐5p and VEGF-A. (b) TCGA data analysis confirmed overexpression of VEGF-A in PDAC. (c) Negative correlation was noticed between miR-150-5p and VEGF-A using StarBase. (d) The scattered plot showed the positive correlation between SNHG10 and VEGF-A using GEPIA analysis of the PDAC TCGA data.


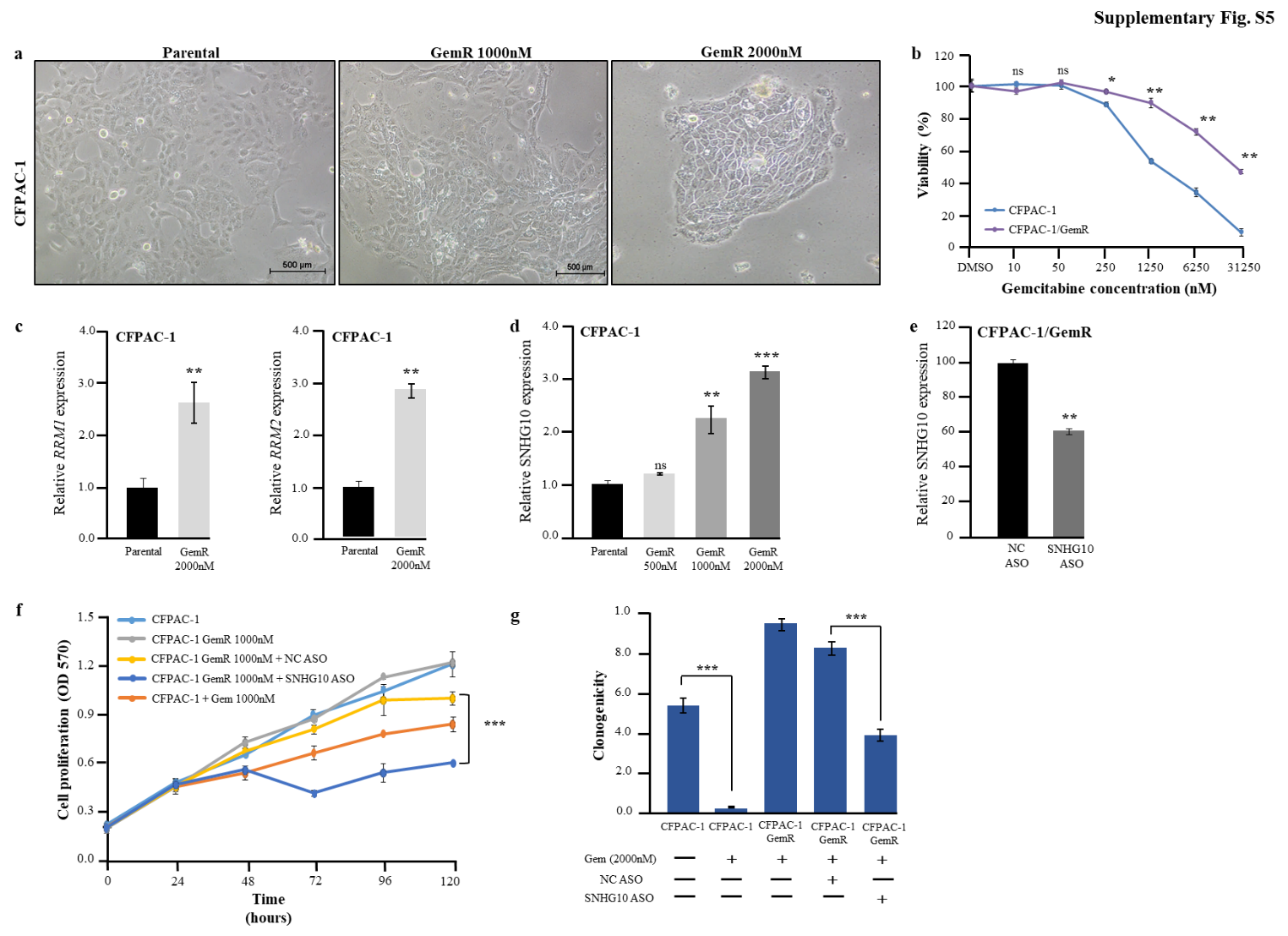


**Supplementary Fig. S5. Downregulation of SNHG10 enhanced the gemcitabine resistance in the in vitro model of gemcitabine-resistant PDAC cells.** (a) Representative images showed morphological changes in gemcitabine-resistant CFPAC-1 cells. (b) MTT confirmed the differential sensitivity of gemcitabine. (c) Expression analysis of gemcitabine resistance-related genes. (d) PCR data displayed the induction of SNHG10 in gemcitabine-resistant cells. (e) qPCR displayed knockdown of SNHG10. (f, g) Proliferation and clonogenic assays demonstrated gemcitabine sensitivity. The results are presented as the means ± SDs; n=3. *p < 0.05, **p < 0.01, ***p < 0.001; two-tailed Student t test.


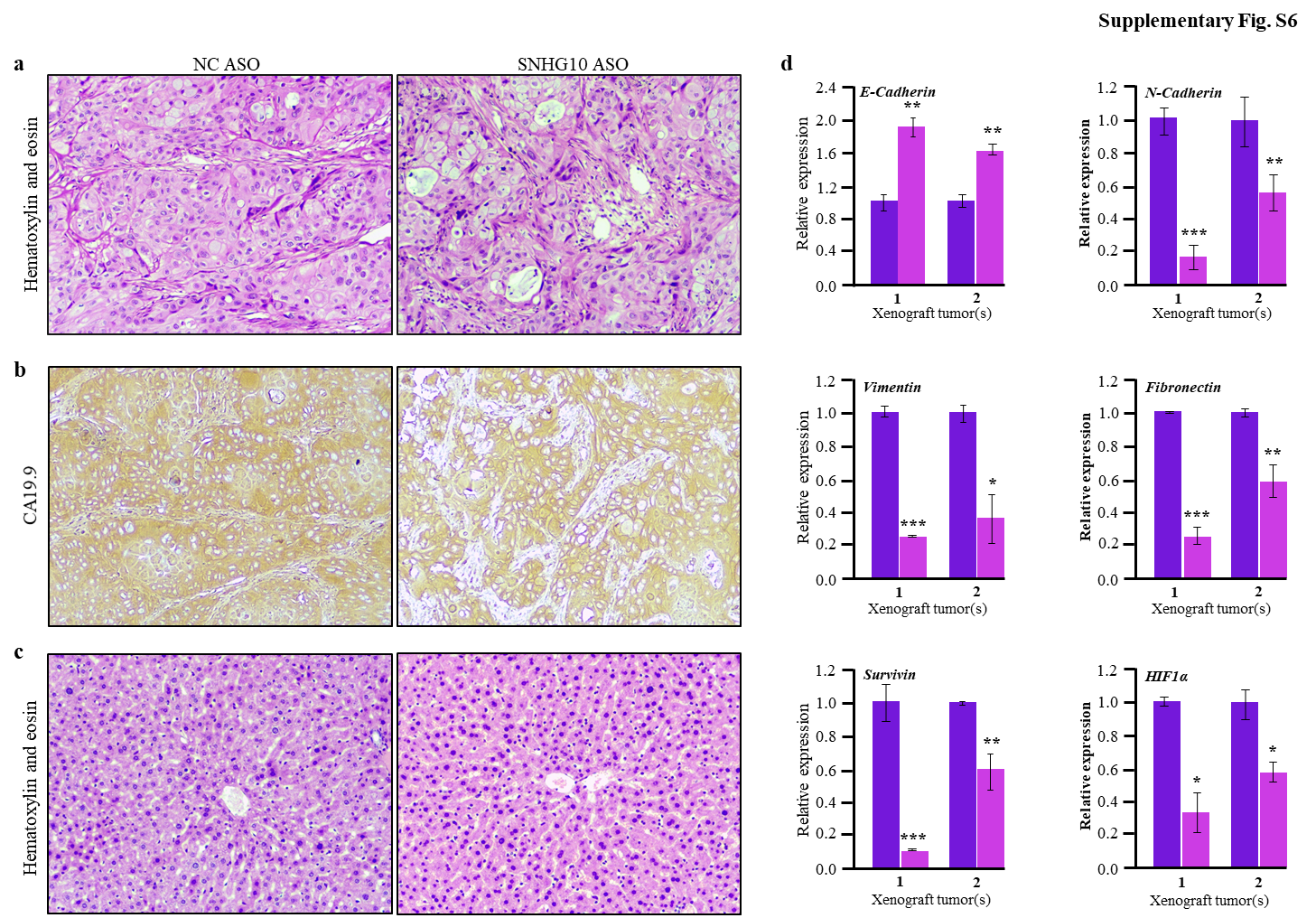


**Supplementary Fig. S6. Effect of SNHG10 ASO treatment in PDAC xenograft model.** (a-c) H&E, and IHC staining of SNHG10 ASO-treated and NC ASO xenograft tumor and liver sections. CA19.9 staining confirms the origin of PDAC. (d) The qRT-PCR data revealed *E-cadherin*, *N-cadherin*, *vimentin*, *fibronectin*, *survivin*, and *HIF-1α* expression in ASO-treated tumors. The results are presented as the means ± SDs; n=3. *p < 0.05, **p < 0.01, ***p < 0.001; two-tailed Student t test.

**Supplementary Table 1:** Details of the quantitative real-time PCR primer

| **Gene Name** | **Forward Primer** | **Reverse Primer** |
| --- | --- | --- |
| SNHG10 | 5’-CAA GCC TCA TCA GGC CCA TT-3’  5’-TGC TCT GCT CTC CCT TGT AC-3’ | 5’-AGT CCA CTG GTC CTG CTC TT-3’  5′-GCG GCT CCA AGA CTA CAG AT-3′ |
| *GAPDH* | 5’-GGA AGG TGA AGG TCG GAG T-3’ | 5’-CCA TGG GTG GAA TCA TAT TGG A-3’ |
| *E-Cadherin* | 5’-TTA CTG CCC CCA GAG GAG GA-3’  5′-AGA ACG CAT TGC CAC ATA CAC TCT C-3′ | 5’-TGC AAC GTC GTT ACG AGT CA-3’  5′-CGG TTA CCG TGA TCA AAA TCT CCA-3′ |
| *N-Cadherin* | 5’-CCG GTT TCA TTT GAG GGC AC-3’ | 5’-TCC CTC AGG AAC TGT CCA T-3’ |
| *Vimentin* | 5’-CTG CCA ACC GGA ACA ATG AC-3’  5’-TGC AGG AGG AGA TGC TTC AG-3’ | 5’-CAT TTC ACG CAT CTG GCG TT-3’  5’-ATT CCA CTT TGC GTT CAA GG-3’ |
| *HIF1a* | 5’ATC CAT GTG ACC ATG AGG AAA TG-3’ | 5’-TCG GCT AGT TAG GGT ACA CTT C-3’ |
| *Survivin*  *(BRIC5)* | 5’-GTT GCG CTT TCC TTT CTG TC-3’ | 5’-CTT TCT CCG CAG TTT CCT CA-3’ |
| *miR-150-5p* | 5’-GTG TCT CCC AAC CCT TGT A-3’ | 5’- GTG CAG GGT CCG AGG T-3’ |
| *U6* | 5’-GTG CTC GCT TCG GCA GCA CAT ATA C-3’ | 5’-AAA AAT ATG GAA CGC TTC ACG AAT TTG-3’ |
| *hENT* | 5’-GCC TGA GCC TGA ACT AGG AG-3’ | 5’-GCC AGA CAG CTT TGT ATC TGT C-3’ |
| *RRM1* | 5’-ACT ATG CTA TCC TGG CAG CC-3’ | 5’-CCG CTC TAG CGT CTT AAA GC-3’ |
| *RRM2* | 5’-AAG AGG CTA CCT ATG GTG AAC G-3’ | 5’-TCC GAT GGT TTG TGT ACC AGG-3’ |
| *VEGF-A* | 5’-GCA CCC ATG GCA GAA GG-3’ | 5’-CTC GAT TGG ATG GCA GTA GCT-3’ |

**Supplementary Table 2:** The sequence of oligonucleotides for SNHG10 siRNA(s), SNHG10 ASO, Negative Control ASO, miR-150-5p inhibitor

| **S. No** | **Name of the oligonucleotides** | **Sequences of the oligonucleotides** |
| --- | --- | --- |
| 1 | SNHG10 siRNA  Smart pool | siSNHG10-1 5′-GCUUGACAAUAUACAGUUA-3′  siSNHG10-2 5′-GCAUAAUUGUUGUUUCAGA-3′  siSNHG10-3 5′-UACUAUUGGUCGUCGGCAA-3′  siSNHG10-4 5′-CAAGAGGGAAGACGACUUU-3′ |
| 2 | SNHG10 ASO | 5′-CTAGAGAAATAATCGC-3′ |
| 3 | Negative control ASO (NC ASO) | 5′-GCTCCCTTCAATCCA-3′ |
| 4 | hsa-miR-150-5p inhibitor | 5’-CACUGGUACAAGGGUUGGGAGA-3’ |

**Note:** siRNA: Small interfering RNA; ASO: Antisense oligonucleotide; **miR:** MicroRNA

**Supplementary Table 3:** The details of antibodies used for the western blotting experiments

| **S. No.** | **Antibodies name** | **Molecular Weight (kDa)** | **Isotype** | **Catalog No.** |
| --- | --- | --- | --- | --- |
| 1 | GAPDH | 37 | Mouse | SC47724 |
| 2 | E-Cadherin (4A2) | 135 | Mouse | CST-14472S |
| 3 | N-Cadherin (13A9) | 140 | Mouse | CST-14215S |
| 4 | Vimentin (VIM; D21H3) | 57 | Rabbit | CST-5741S |
| 5 | p-21 Waf 1/Cip1 (12D1) | 21 | Rabbit | CST-2947 |
| 6 | CDK-4 (D9G3E) | 30 | Rabbit | CST-12790T |
| 7 | CDK-6 (DCS83) | 36 | Mouse | CST-3136T |
| 8 | Cyclin B1(V152) | 55 | Mouse | CST-4135S |
| 9 | Cyclin D1(92G2) | 34 | Rabbit | CST-2978T |
| 10 | Aurora A/AIK1 (1G4) | 48 | Rabbit | CST-4718P |
| 11 | Aurora B/AIM1 | 40 | Rabbit | CST-3094P |
| 12 | Survivin (71G4B7) | 16 | Rabbit | CST-2808T |
| 13 | VEGF-A (A-20) | 42 | Rabbit | SC-152 |
| 14 | p-AKT (Ser473) (D9E) | 60 | Rabbit | CST-4060S |
| 15 | Pan AKT (40D4) | 60 | Mouse | CST-2920S |
| 16 | p-p44/42(pERK1/2) (Thr202/Tyr204) (D13.14.4E) | 42, 44 | Rabbit | CST-4370S |
| 17 | p44/42 MAPK (ERK1/2) (137F5) | 42, 44 | Rabbit | CST-4695S |
| 18 | Phospho-EGFR (Tyr1068) | 175 | Rabbit | CST- 2234S |
| 19 | EGF Receptor (EGFR; D38B1) | 175 | Rabbit | CST-4267S |
| 20 | Phospho-mTOR (Ser2448)(D9C2) | 289 | Rabbit | CST-5536T |
| 21 | mTOR (7C10) | 289 | Rabbit | CST-2983T |
| 22 | Phospho c-Met (Tyr1234/1235) | 145 | Rabbit | CST-3077T |
| 23 | c-Met Antibody | 145 | Rabbit | CST-4560S |
| 24 | VEGF Receptor 2 (55B11) | 210,230 | Rabbit | CST-2479 |
| 25 | Anti-Mouse IgG, HRP Linked | - | Horse | 7076P2 |
| 26 | Anti-Rabbit IgG, HRP Linked | - | Goat | 7074P2 |

**Figure 2c: Original Western Blots**

**
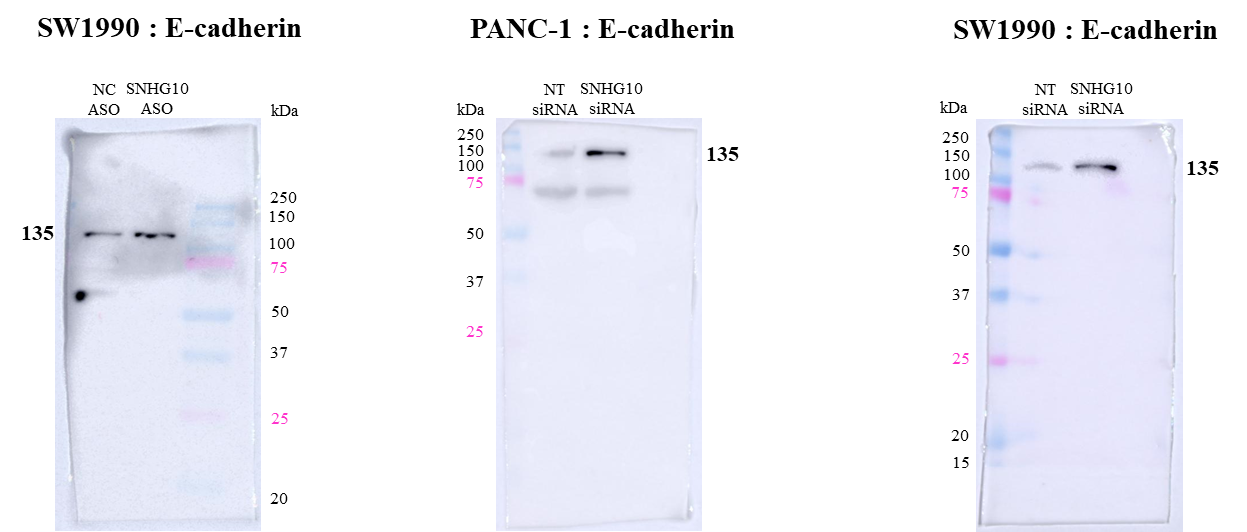
**

**
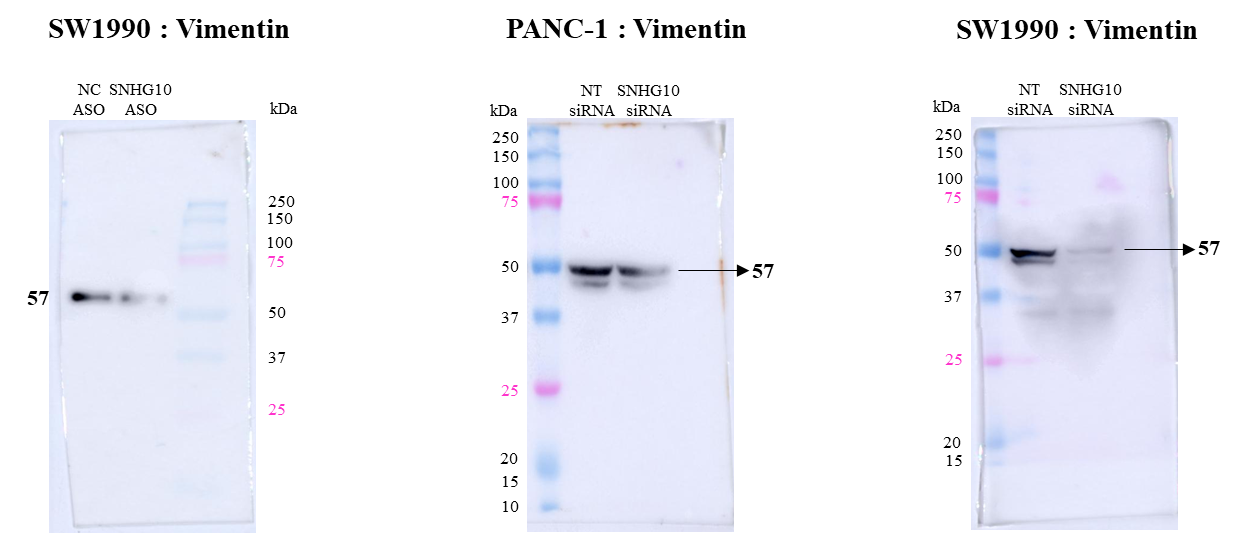
**

**
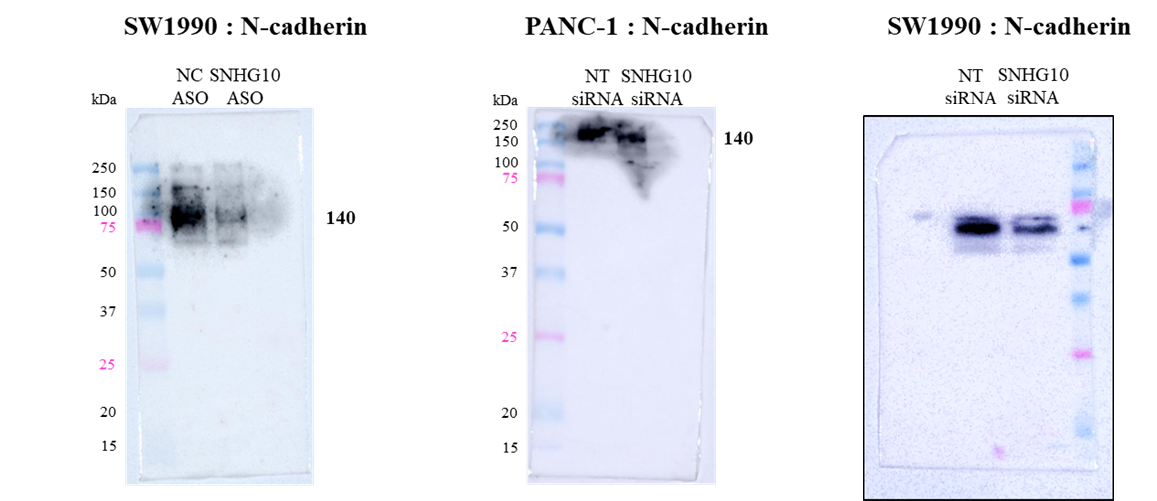
**

**
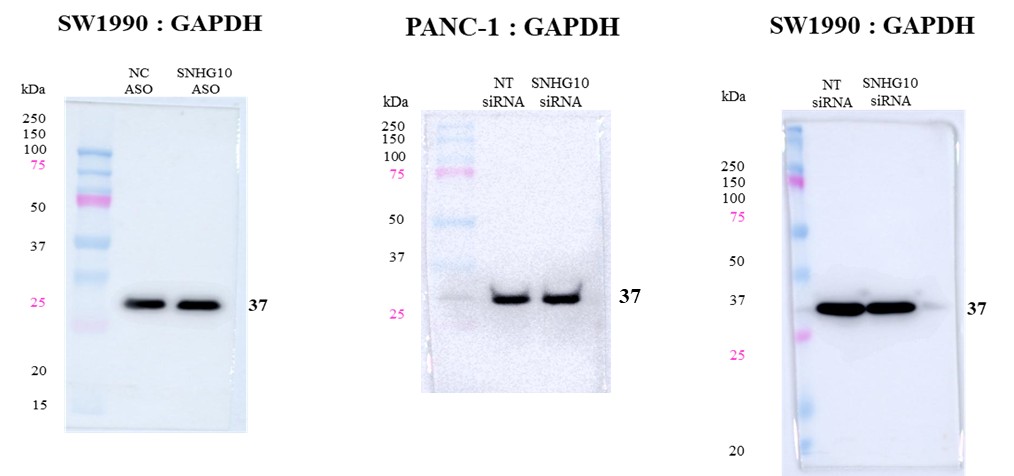
**

**Figure 3e: Original Western Blots**

**
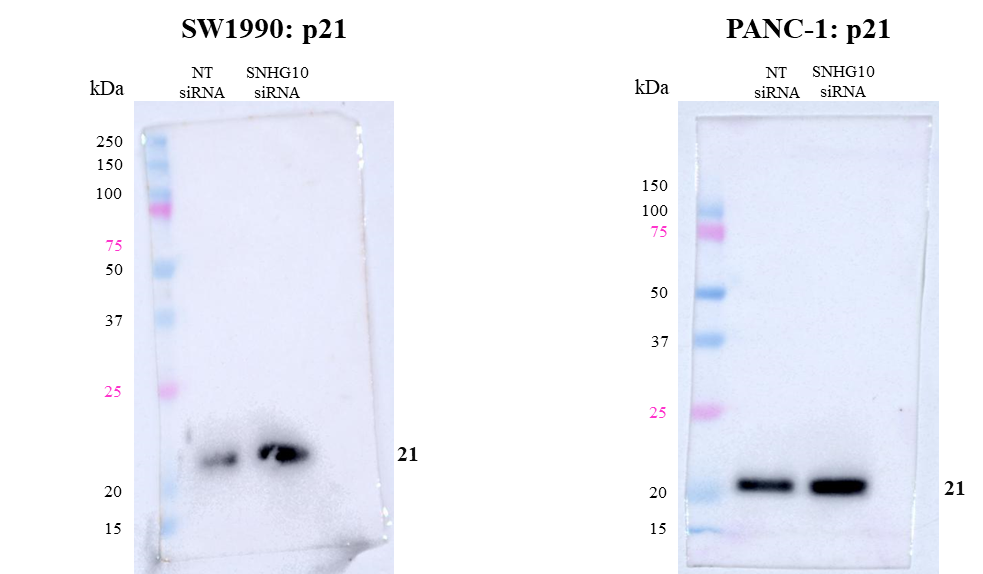
**

**
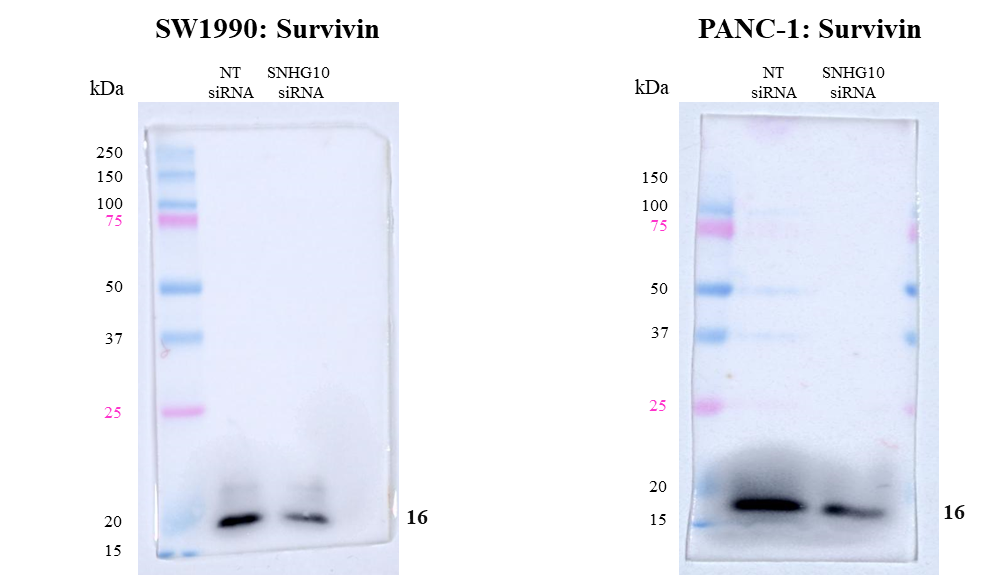
**

**
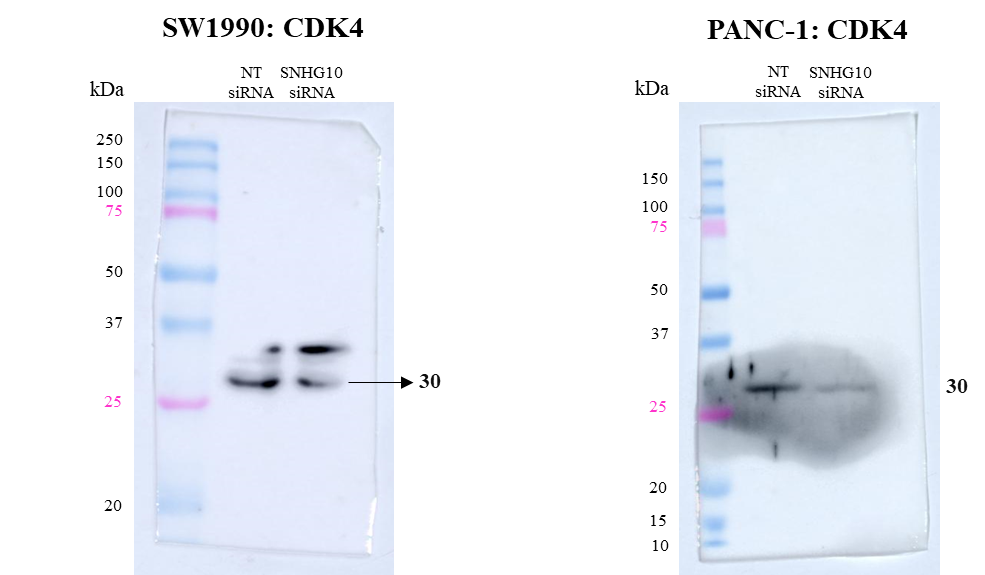
**

**
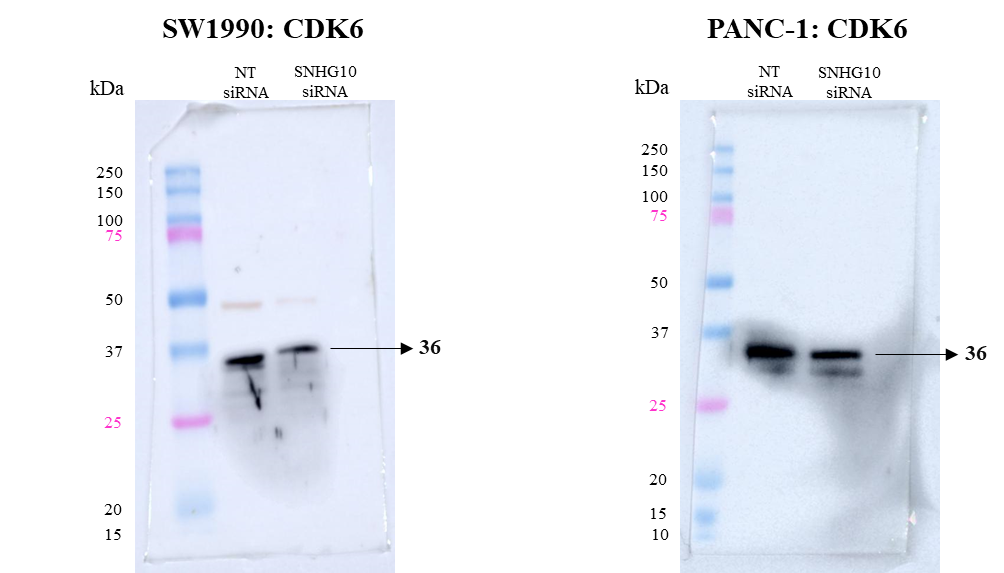
**

**
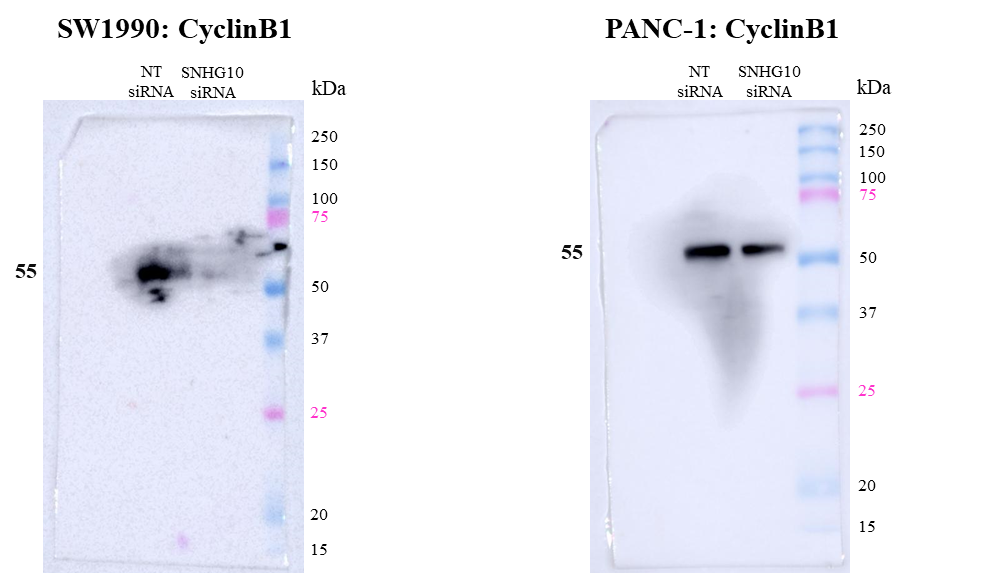
**

**
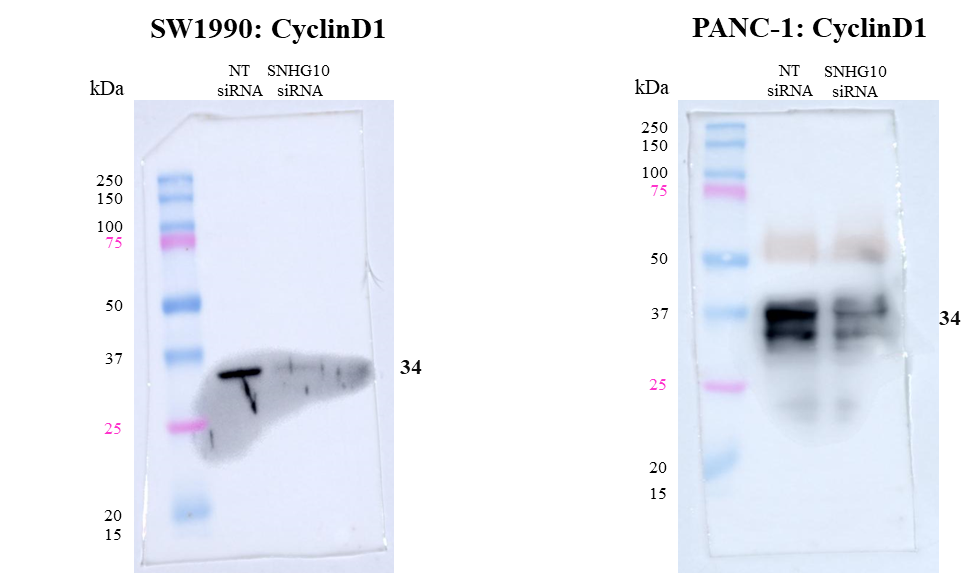
**

**
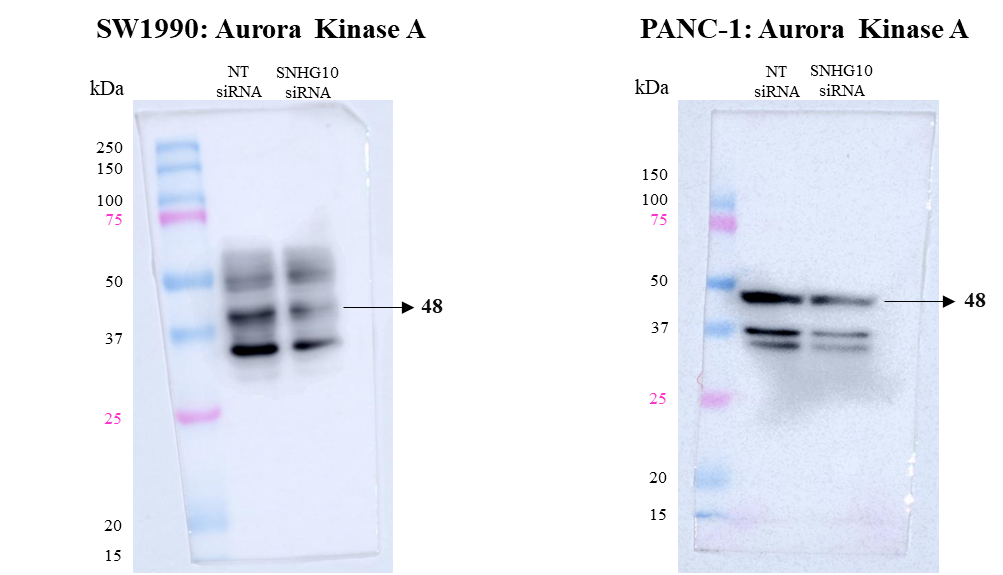
**

**
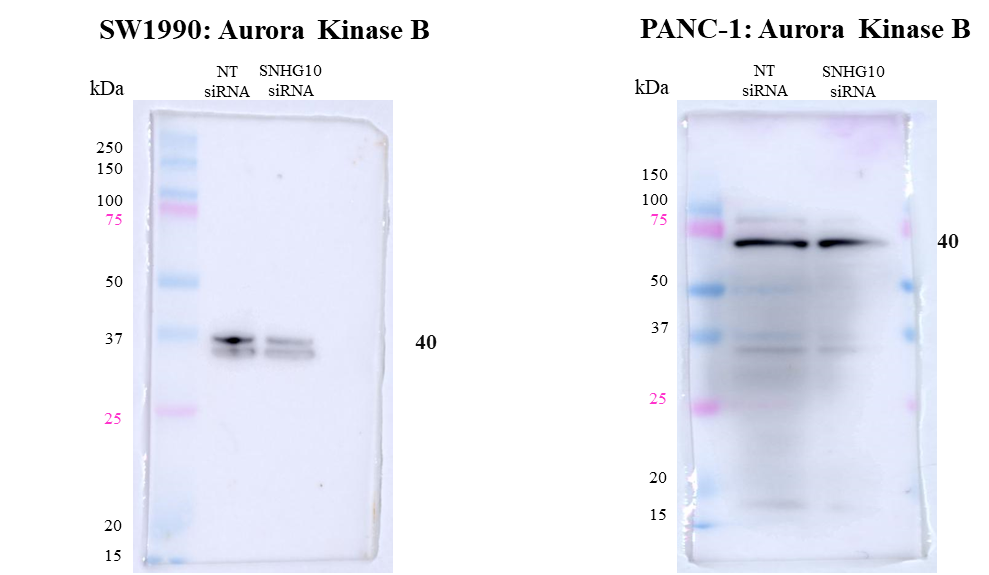
**

**
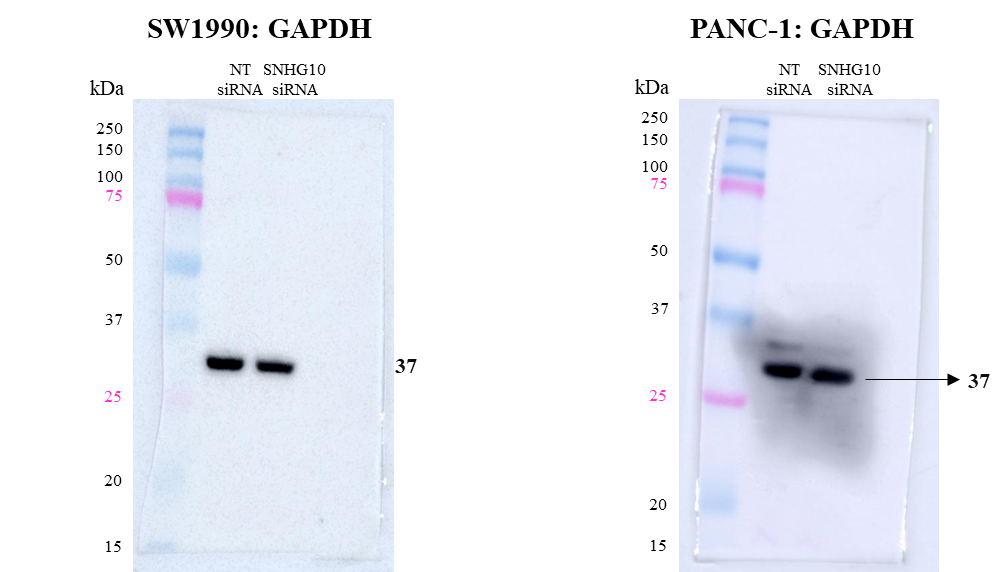
**

**Figure 4h: Original Western Blots**

**
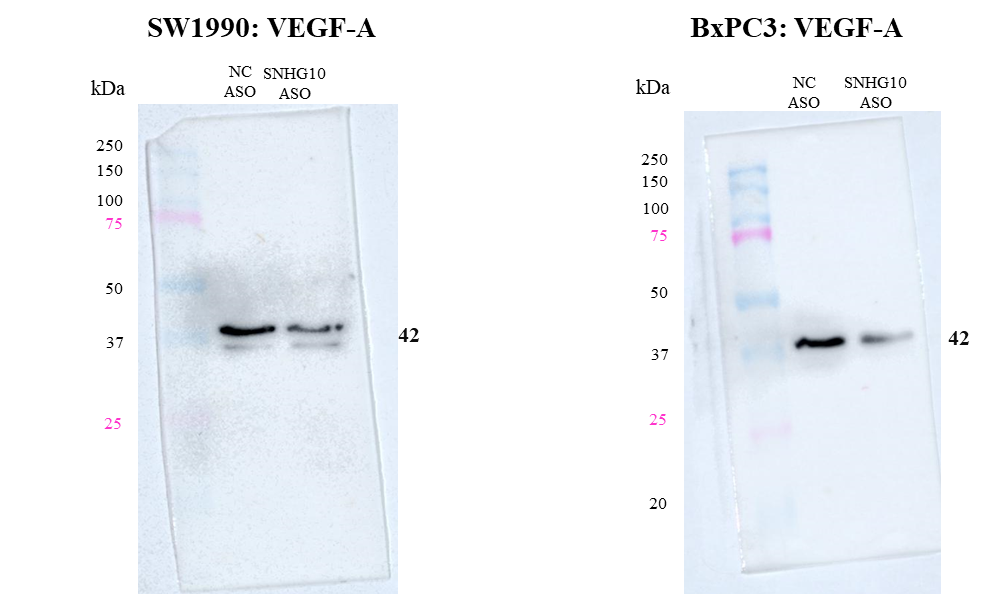
**

**
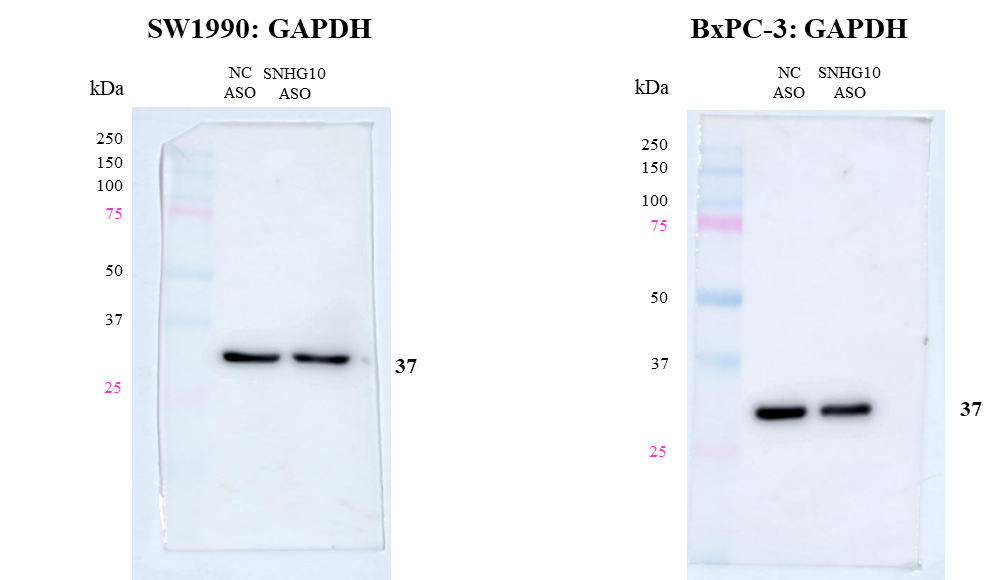
**

**Figure 4n: Original Western Blots (BxPC-3 cell line)**

**
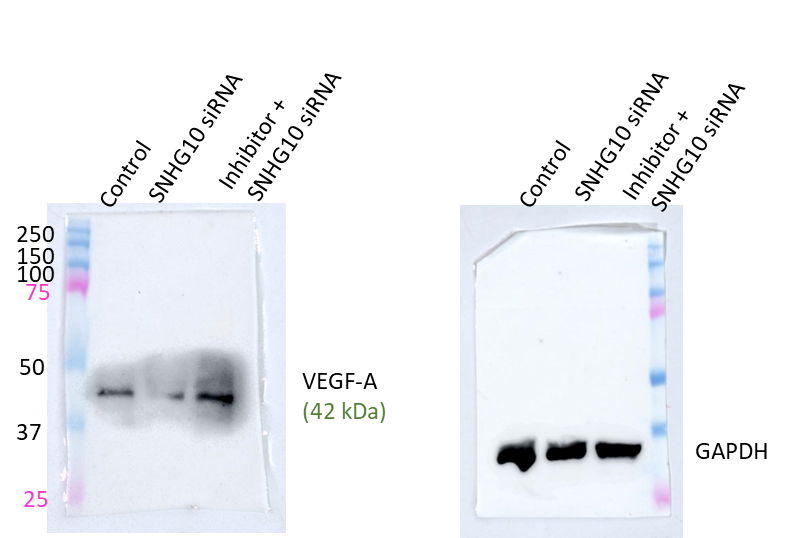
**

**Figure 5a: Original Western Blots**

**
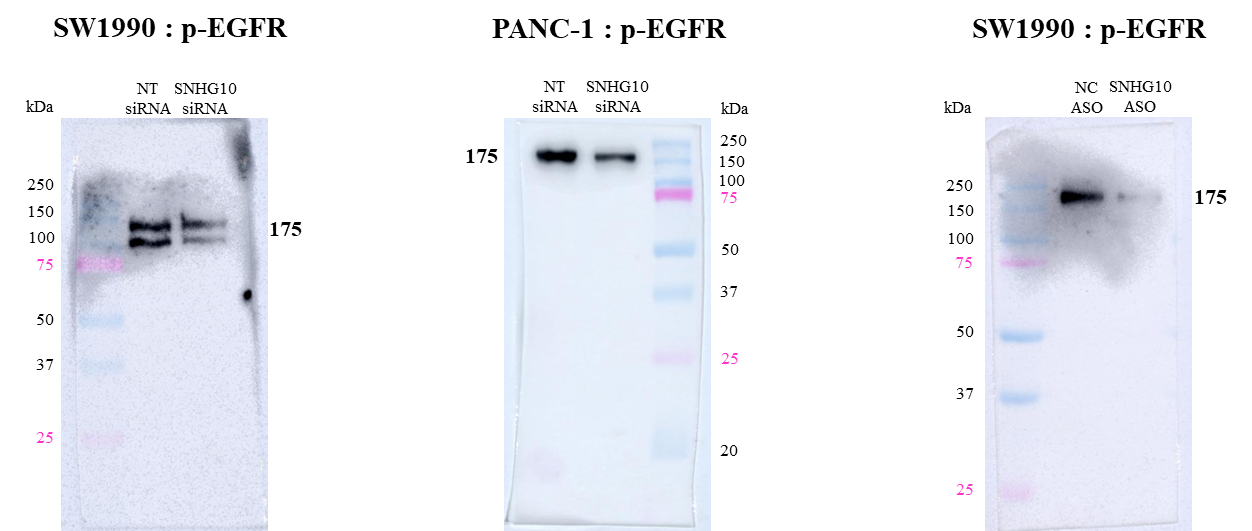
**

**
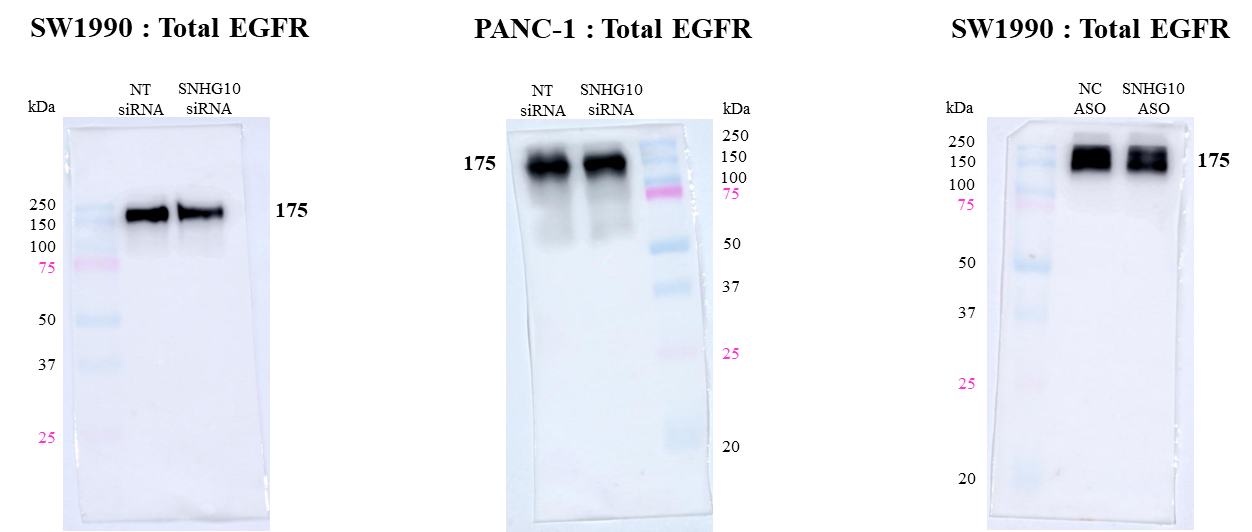
**

**
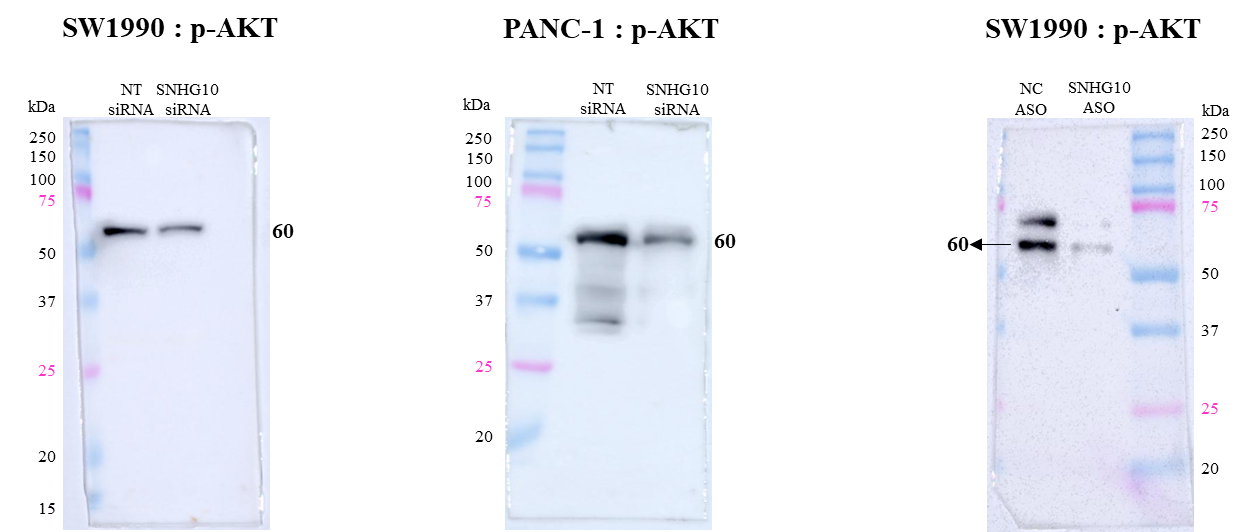
**

**
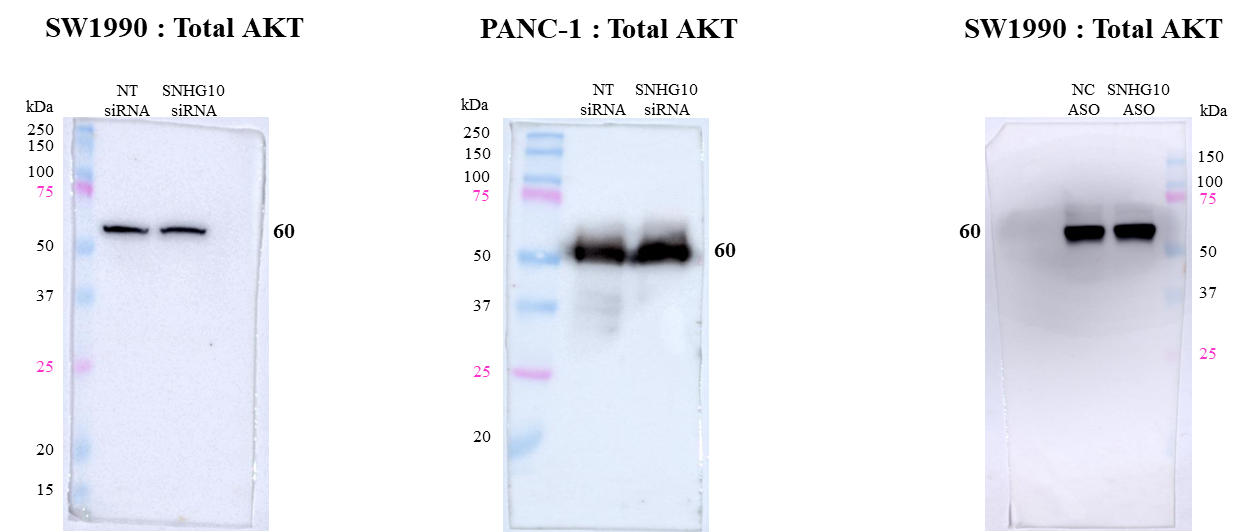
**

**
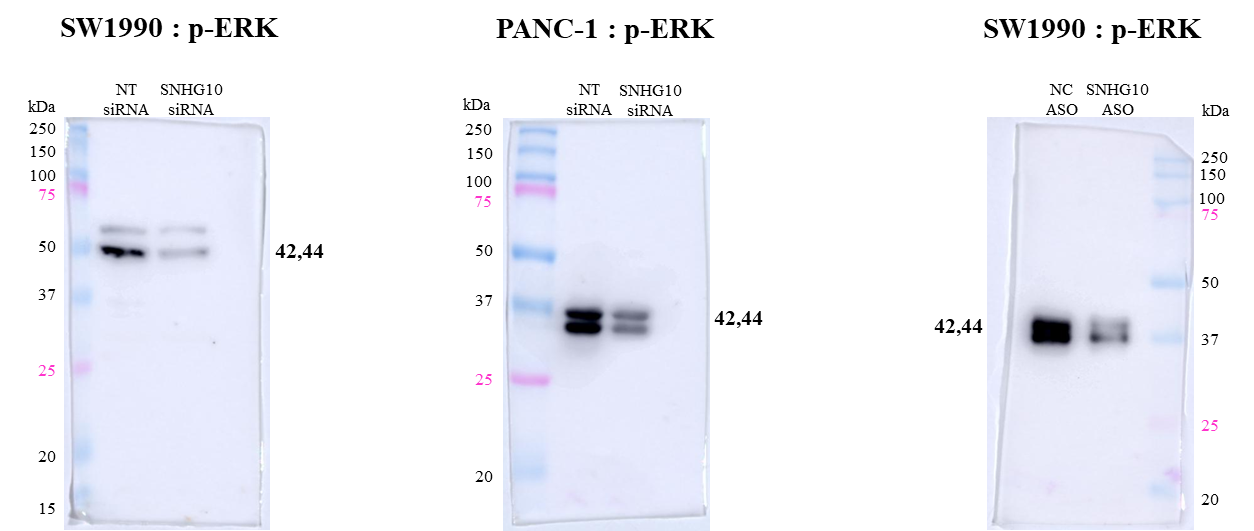
**

**
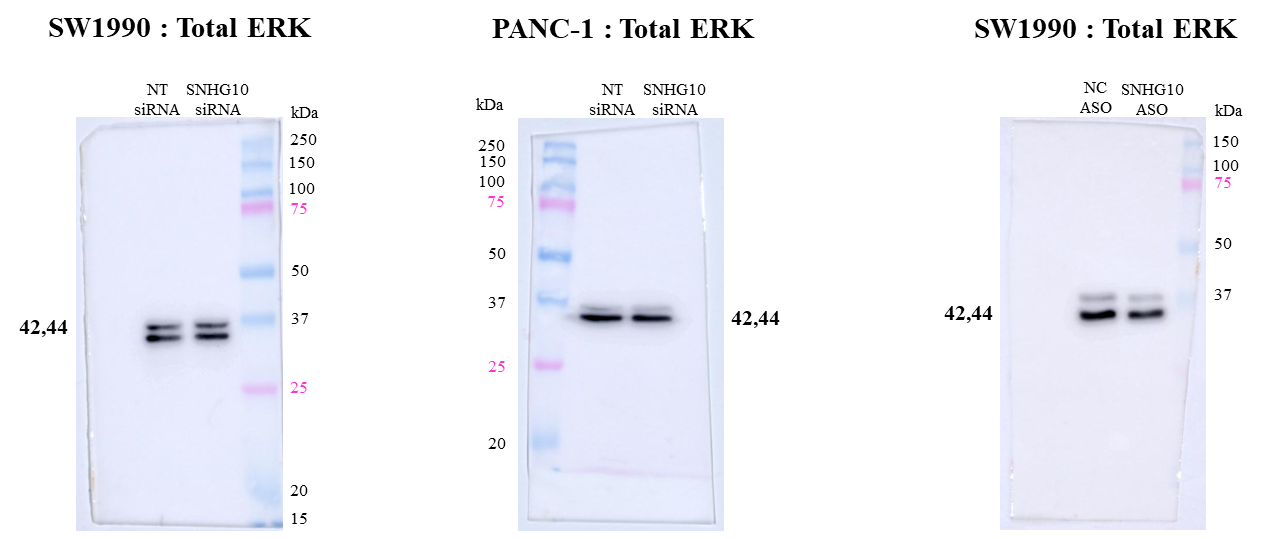
**

**
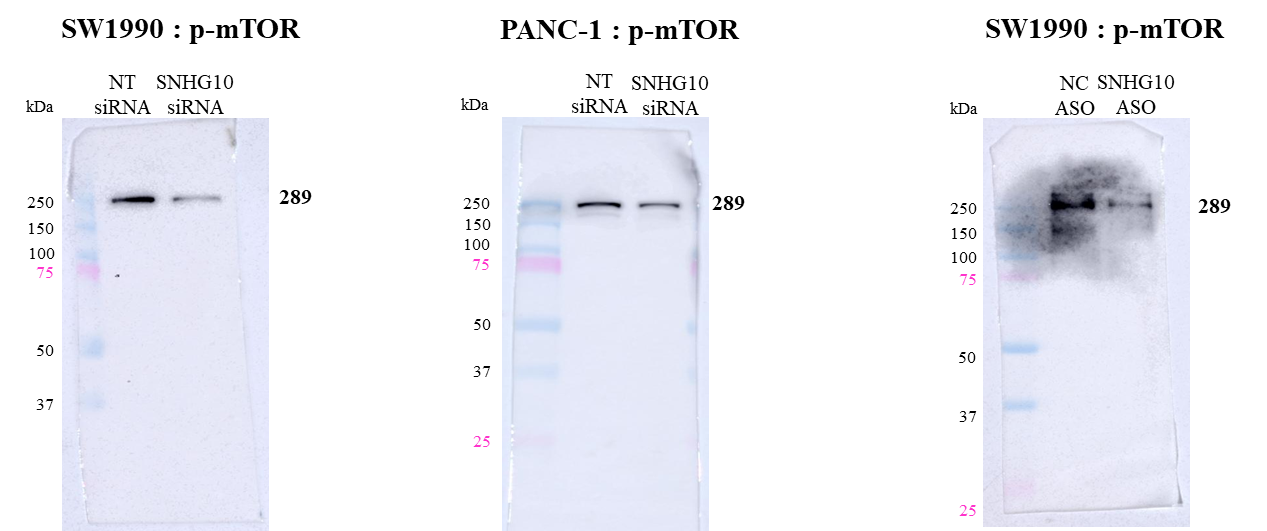
**

**
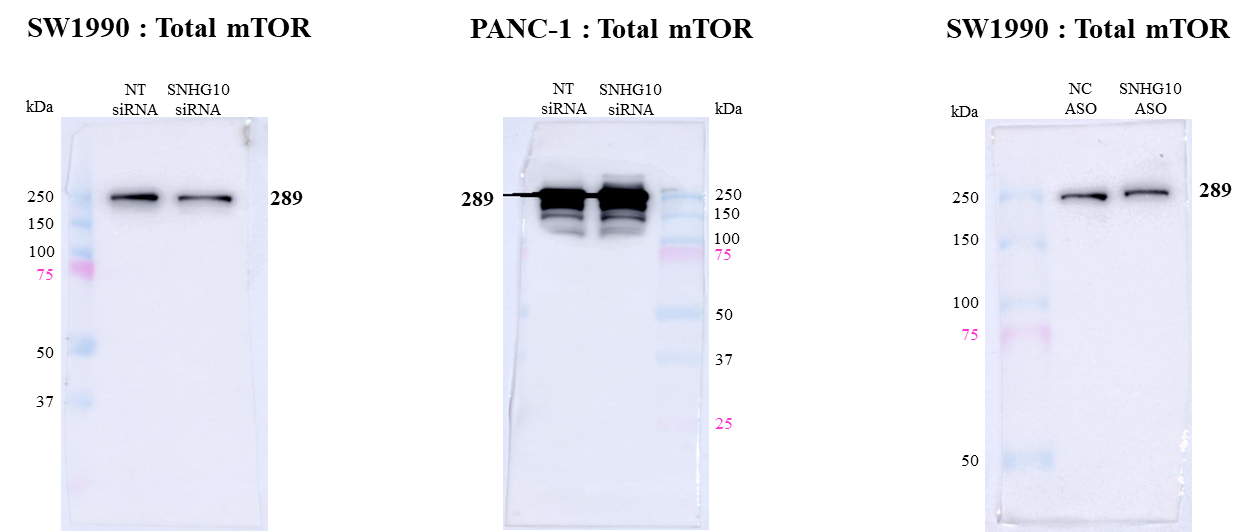
**

**
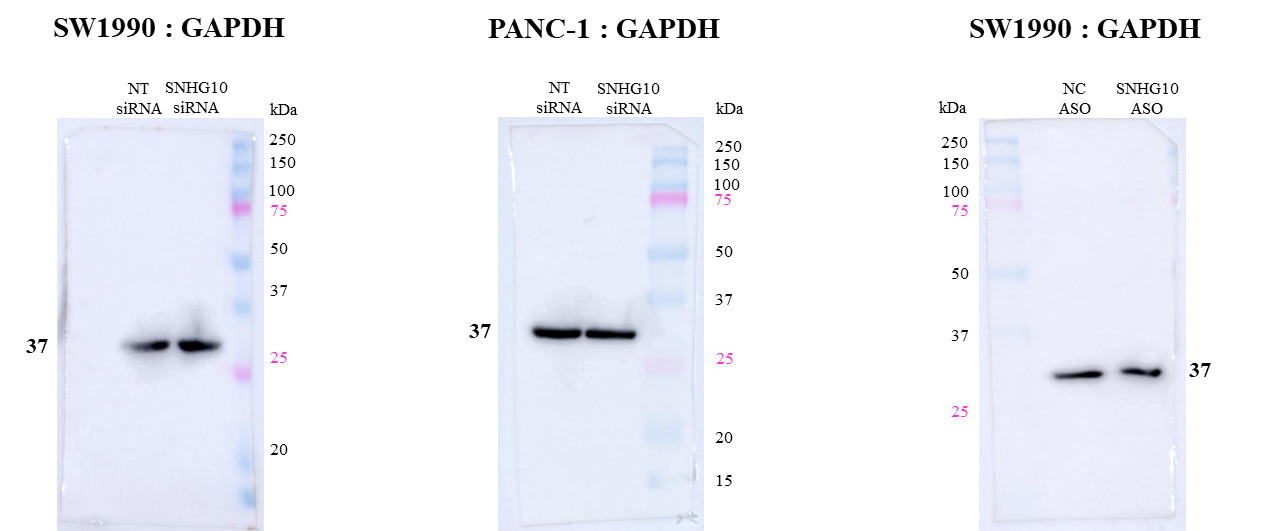
**

**
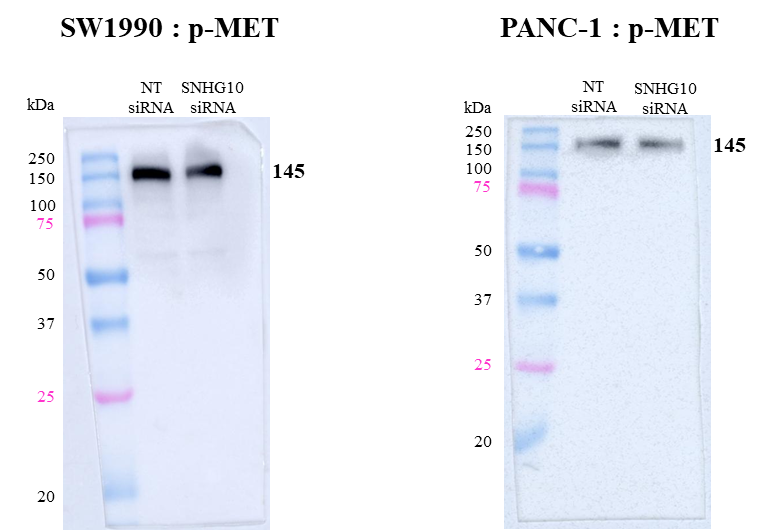
**

**
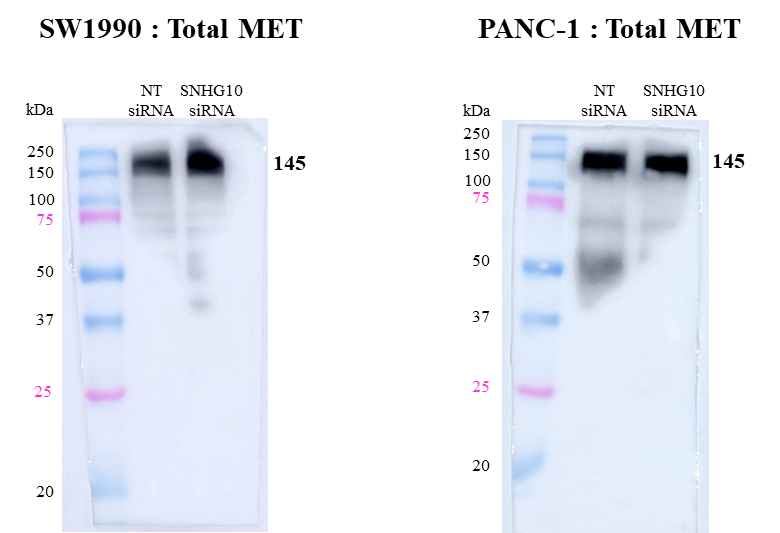
**

**
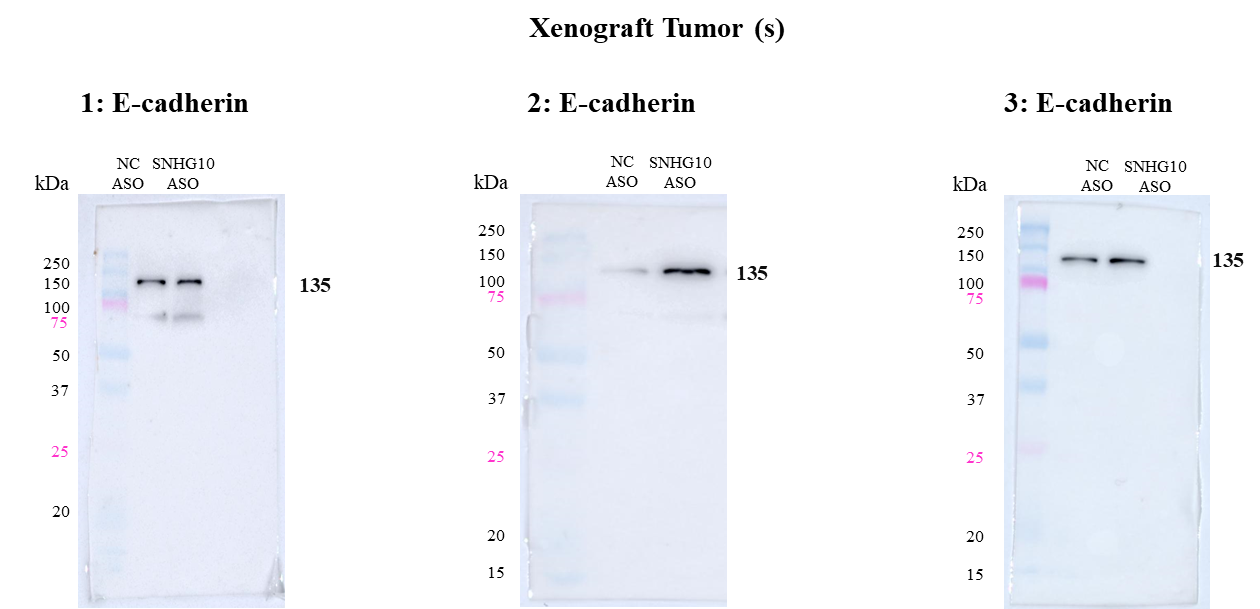
Figure 8a: Original Western Blots**

**
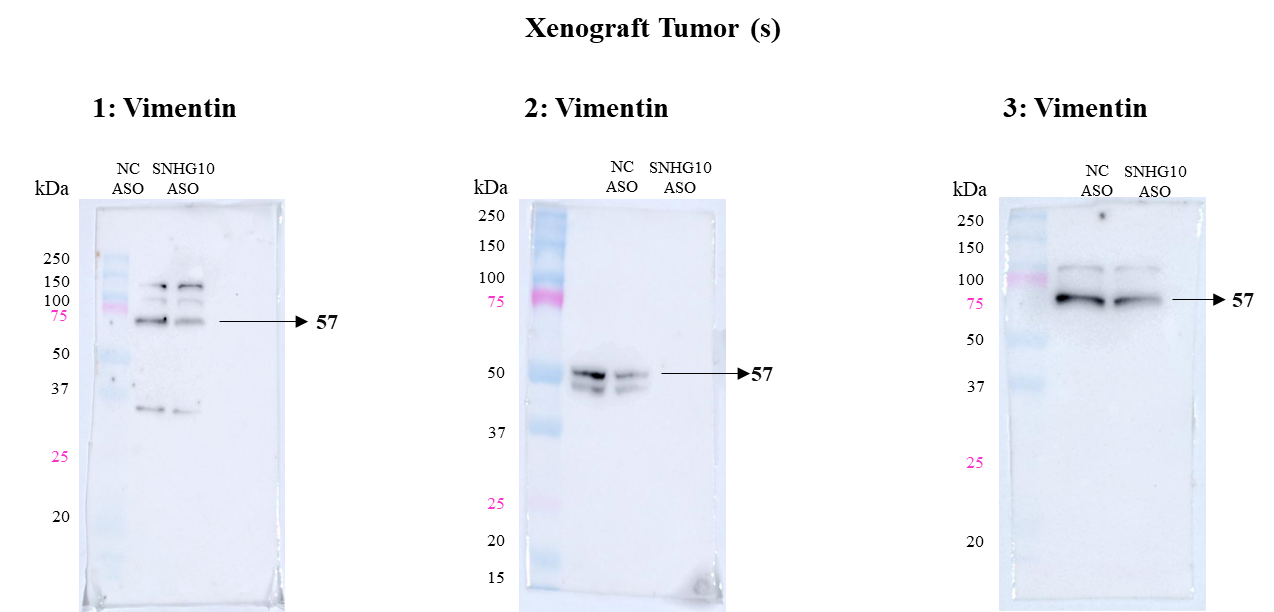
**

**
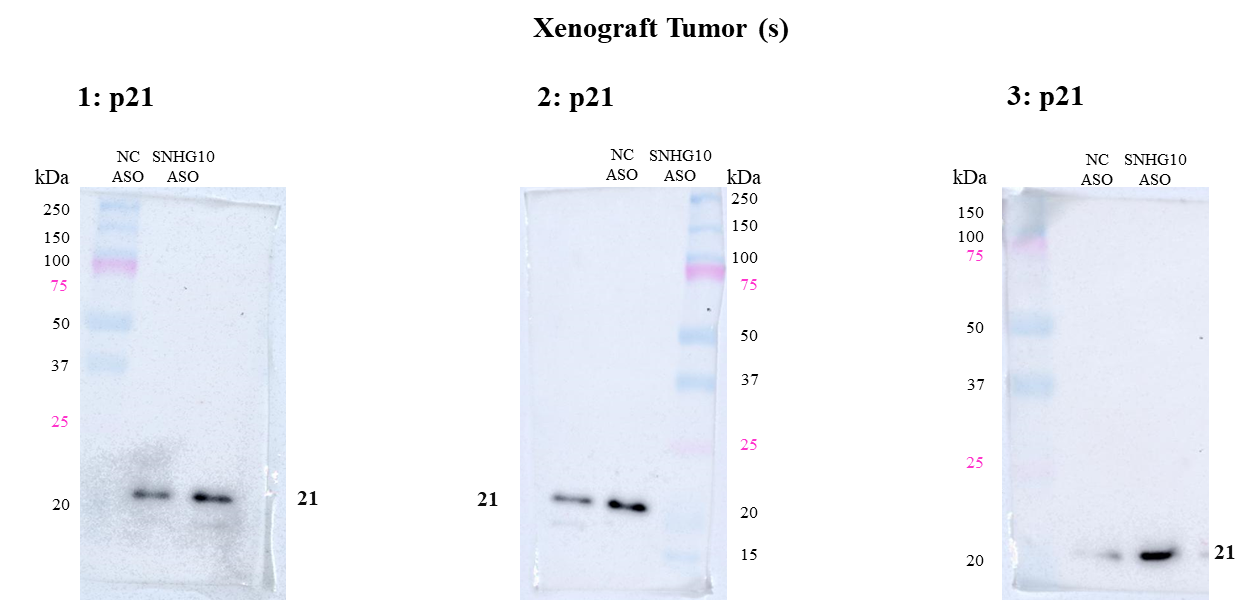
**

**
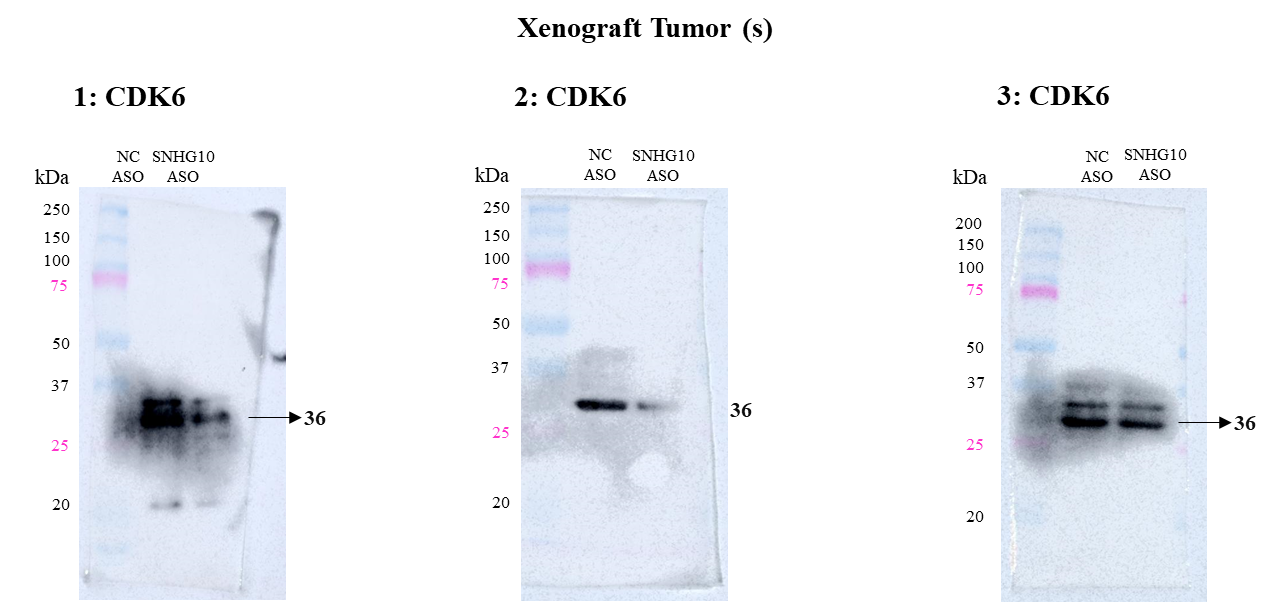
**

**
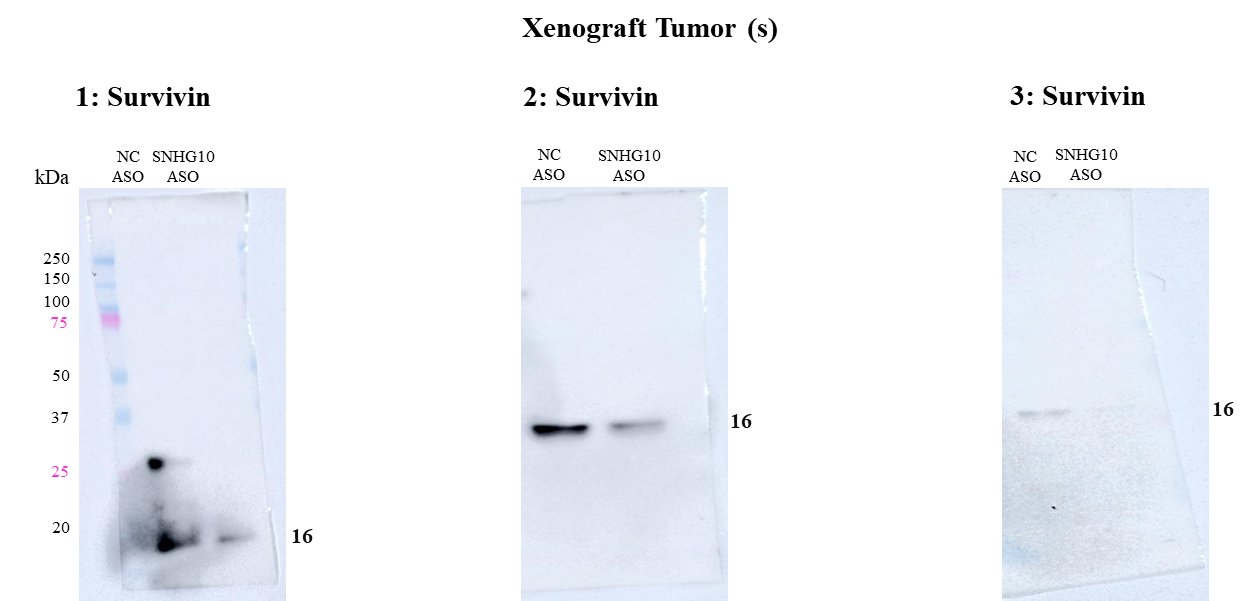
**

**
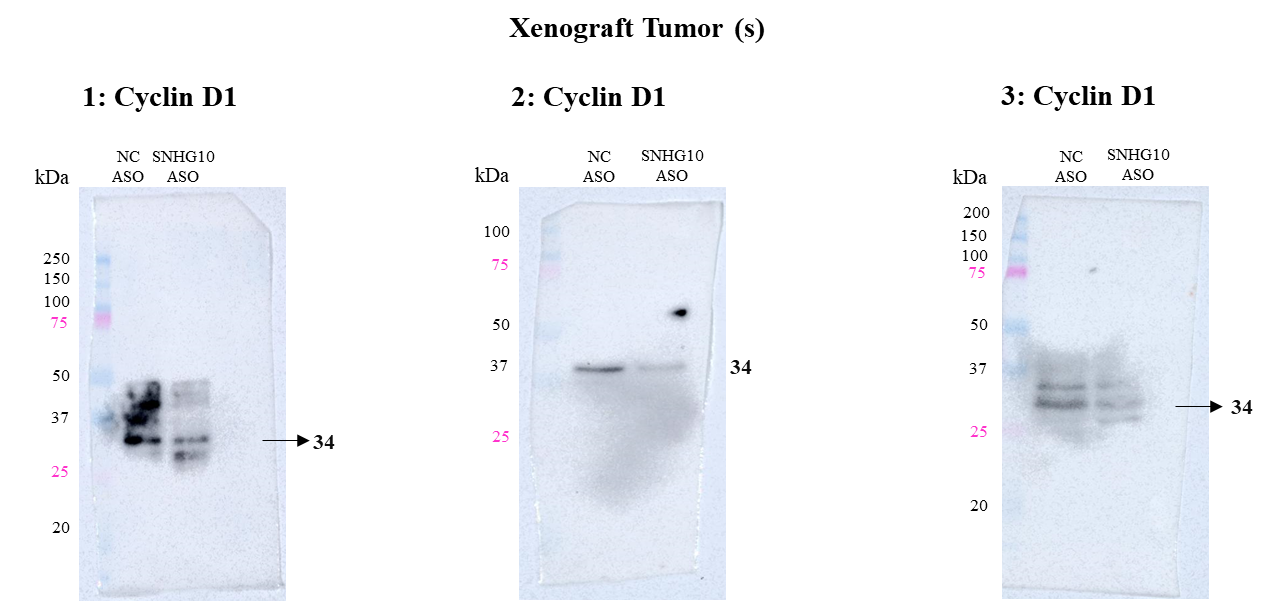
**

**
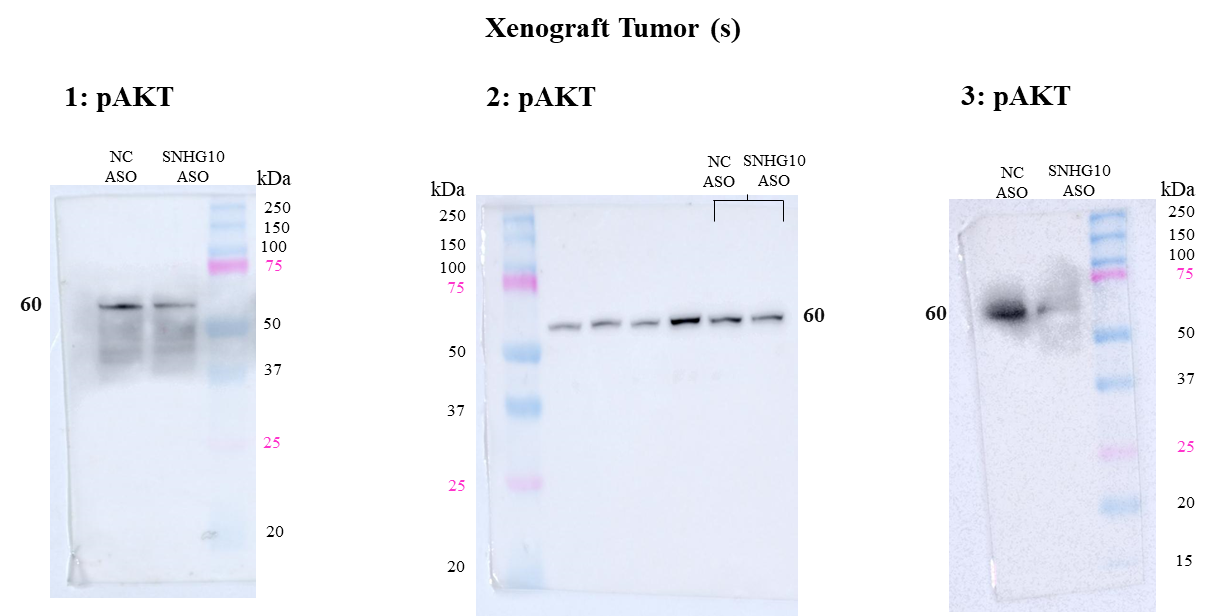
**

**
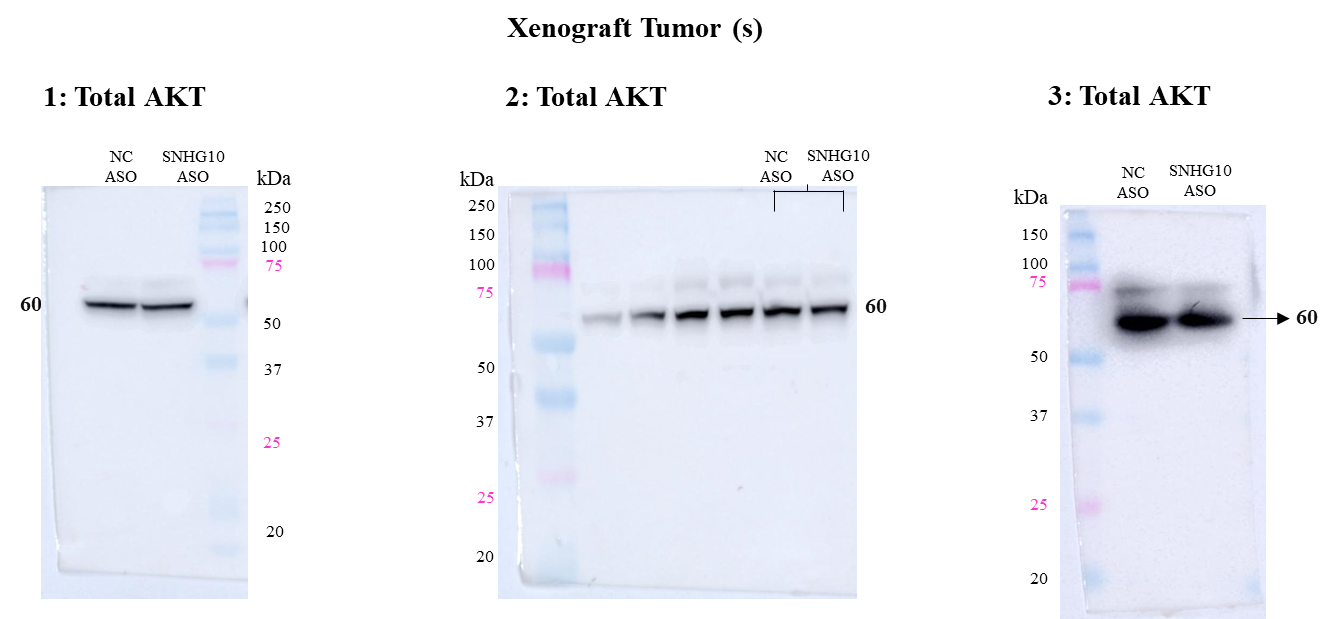
**

**
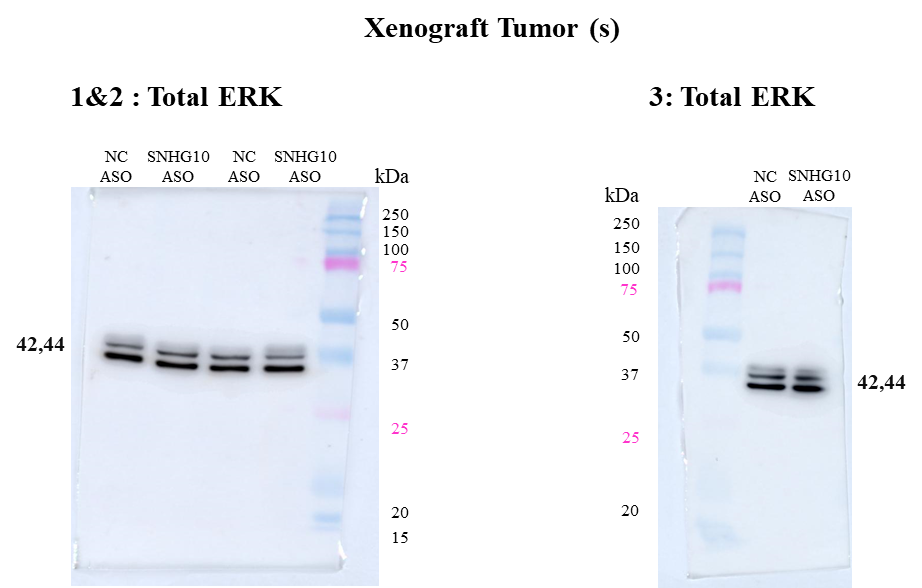
**

**
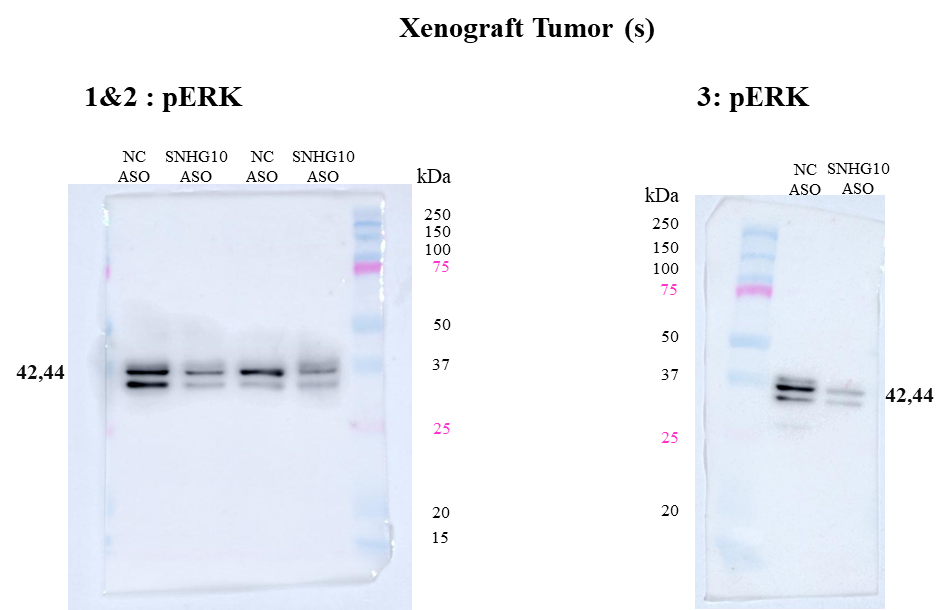
**

**
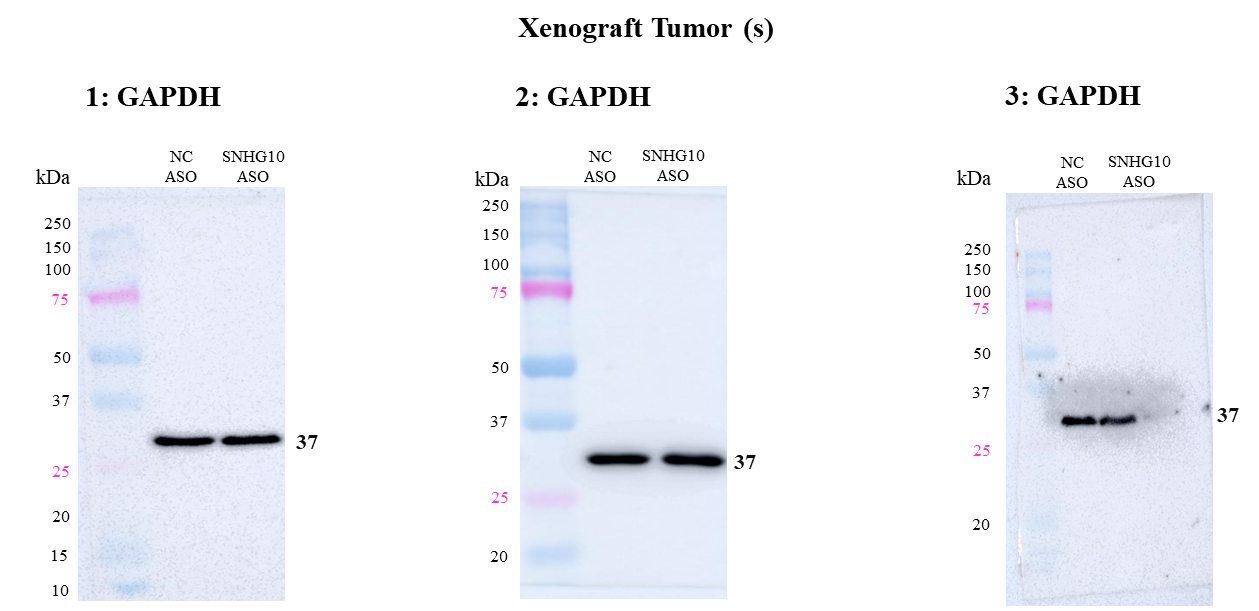
**

**Fig. S4e: Original Western Blots (SW1990)**

**
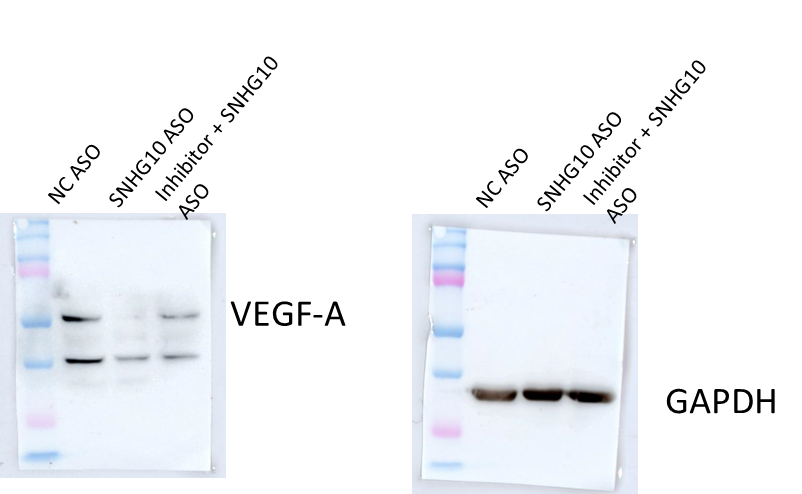
**
